# Back to basics: A mediation analysis approach to addressing the fundamental questions of integrated care evaluations

**DOI:** 10.1002/hec.4713

**Published:** 2023-05-26

**Authors:** David G. Lugo‐Palacios, Jonathan M. Clarke, Søren Rud Kristensen

**Affiliations:** ^1^ Centre for Health Policy Institute of Global Health Innovation Imperial College London London UK; ^2^ Department of Health Services Research & Policy London School of Hygiene & Tropical Medicine London UK; ^3^ EPSRC Centre for Mathematics of Precision Healthcare Imperial College London London UK; ^4^ Danish Centre for Health Economics (DaCHE) University of Southern Denmark Odense Denmark

**Keywords:** ambulatory care sensitive admissions, concentration index, integrated care, mediation analysis, outpatient care referrals

## Abstract

Health systems around the world are aiming to improve the integration of health and social care services to deliver better care for patients. Existing evaluations have focused exclusively on the impact of care integration on health outcomes and found little effect. That suggests the need to take a step back and ask whether integrated care programmes actually lead to greater clinical integration of care and indeed whether greater integration is associated with improved health outcomes. We propose a mediation analysis approach to address these two fundamental questions when evaluating integrated care programmes. We illustrate our approach by re‐examining the impact of an English integrated care program on clinical integration and assessing whether greater integration is causally associated with fewer admissions for ambulatory care sensitive conditions. We measure clinical integration using a concentration index of outpatient referrals at the general practice level. While we find that the scheme increased integration of primary and secondary care, clinical integration did not mediate a decrease in unplanned hospital admissions. Our analysis emphasizes the need to better understand the hypothesized causal impact of integration on health outcomes and demonstrates how mediation analysis can inform future evaluations and program design.

## 1 INTRODUCTION

Integrated care has risen to the top of the healthcare agenda in many high income countries in response to populations living longer with chronic conditions. The shifting demographics have led to a call for health system redesign with greater emphasis on long term care continuity (Hughes et al., 2020; Nolte & Pitchforth, 2014). Economics research on the topic dates back at least 20 years when the potential impact of horizontal and vertical integration of care was studied prompted by the rise of managed care (Cutler et al., 2000; Gaynor & Haas‐Wilson, 1999) and later accountable care organisations (ACOs) in the United States (Frandsen & Rebitzer, 2014; McWilliams et al., 2016). Important questions raised by this literature are whether horizontal and vertical integration is associated with efficiency increases and to which extent care integration is anti‐competitive (Cebul et al., 2008; Gaynor, 2006). A related literature has focused more on the causes and consequences of care fragmentation, framing the question as a trade‐off between specialization and coordination (Agha et al., 2019; Becker & Murphy, 1992).

The long‐standing policy interest in care integration has led to a range of experiments with integrating care in countries with different institutional settings including Australasia, Europe and North America. So far, evaluations have focused on the impact of integrated care on health care use, quality and costs (Hughes et al., 2020). However, although the idea of integrating care seems an intuitive way to address the perceived problem of care fragmentation and lack of continuity, the evidence so far does not convincingly demonstrate integrated care to be the panacea policy makers often expect (Kumpunen et al., 2020).

While existing evaluations have mostly focused on the impact of integration programmes on health outcomes, the mixed findings suggest the need to take a step back and address two more fundamental questions. Firstly, are integrated care models in fact associated with greater clinical integration? This question was already posed in response to the first wave of health maintenance organisations (HMOs) in the US (Burns & Pauly, 2002; Cebul et al., 2008), but to this day remains unresolved, presumably due to the inherent difficulty in addressing the question in empirical research. Secondly, is greater clinical integration in fact associated with improved outcomes? The policy focus on care integration assumes that this is the case, but to our knowledge, this assumption does not build on specific evidence on care integration as a mechanism related to the intended outcomes. Without a profound understanding of these two questions, it is difficult to see how future care reorganisation can be designed to reach the desired outcomes.

This paper aims to shed light on these essential questions by asking how, rather than if integrated care works. Specifically, we use mediation analysis to assess the extent to which an integrated care program in the English National Health Service (NHS) led to greater clinical integration and, in turn, whether greater integration was associated with improved health outcomes for patients.

To address these questions, we re‐evaluate the integrated primary and acute care systems (PACS) which is part of the Vanguards program introducing New Care Models in the English NHS from 2015 to 2018. The impact of the Vanguards program (of which PACS is a subset) was previously studied by Morciano et al. (2020). The analysis by Morciano et al. focused on the direct impact of PACS (analyzed jointly with another Vanguard type known as multispecialty community providers) on emergency admissions and bed days. The authors found no effect on either outcome overall, and when examined individually and by year, only on emergency admissions in the third year of the program. In contrast, our analysis does not in the first instance aim to evaluate the impact of PACS on outcomes directly. Rather, the aim of our analysis is to understand whether PACS affected clinical integration per se, and whether greater integration was causally associated with fewer emergency admissions.

General Practitioners (GPs) play a crucial role in the integration of primary and secondary care. From their referral patterns, we can infer whether GPs changed behavior after the introduction of PACS. Tighter integration is expected to lead to a greater concentration in referrals to the PACS partner hospital. We, therefore, posit that one aspect of clinical integration of care can be measured using the concentration of general practice referrals to specialist outpatient hospital care.

Mediation analysis aims at evaluating the causal mechanisms through which an intervention affects an outcome of interest. The analysis aims to disentangle the total treatment effect into an indirect effect operating through one or several intermediate variables (mediators), and the direct effect which includes any causal mechanism not operating through the mediators of interest. This method has previously been used in psychology and sociology to study, for example, the effects of ethnicity‐based media cues on immigration preferences (Brader et al., 2008) and, more recently in economics, to analyze the effect of less accommodating caseworkers counselling on the successful placement of unemployed workers (Huber et al., 2017) and to investigate the causal pathways through which payment for performance schemes influence maternal care outcomes (Anselmi et al., 2017).

Our mediation analysis approach begins by estimating whether PACS increased clinical integration of primary and secondary care. To that end, we calculate Herfindahl‐Hirschman style concentration indexes (HHI) of outpatient referrals for each practice in the PACS areas and a set of comparison areas. An increase in the HHI suggests that practices concentrated their outpatient referrals among fewer hospitals which we interpret as an indicator of increased clinical integration. In a second step, we examine the impact of the PACS on the rate of ambulatory care sensitive emergency admissions (ACSAs) at practice level and, by conditioning on the variation in the measure of integration of care, we are able to decompose the total policy effect in the indirect effect (through a change in the integration of primary and secondary care) and the direct effect (through all other routes encouraged by the PACS) (Baron & Kenny, 1986; MacKinnon et al., 2007).

While we find evidence to suggest that PACS did in fact increase the clinical integration of care, we do not find strong evidence to support the hypothesis that the integration of primary and secondary care is causally linked to fewer emergency admissions for ambulatory care sensitive conditions. Our analysis therefore confirms the findings of the previous analysis by Morciano et al. and questions whether greater clinical integration could in fact be expected to reduce ambulatory care sensitive emergency admissions.

We make three important contributions to the literature on care integration. Firstly, we highlight the lack of empirical support for the assumed causal relationship between care integration and desired outcomes such as emergency admission to hospital. Secondly, we suggest and demonstrate a mediation analysis approach to assess the impact of specific elements of care integration on outcomes of integrated care interventions. Finally, we suggest the use of GP's concentration of outpatient referrals to measure clinical care integration.

The paper proceed as follows. Section 2 introduces the approaches to care integration in the English NHS and PACS in particular. Section 3 presents the methodology and data including our measure of integration and our outcome measure. Section 4 presents the results and Section 5 concludes.

## 2 CASE STUDY: INTEGRATED PRIMARY AND ACUTE CARE SYSTEMS IN THE ENGLISH NATIONAL HEALTH SERVICE

The English NHS began experimenting with better care integration with the launch of integrated care pilots in 2009–2011 followed by the integrated care pioneers program from 2013 to 2017. In 2015, the NHS Vanguards program launched across England aiming to break down the traditional barriers between health and care organisations by introducing New Care Models to establish more personalized and coordinated health services for patients (NHS, 2016b; Starling, 2017). By September 2016 there was a total of 50 Vanguards classified into five types of models: integrated PACS, multispecialty community providers, enhanced health in care homes, urgent and emergency care, and acute care collaborations (NHS, 2016b). The PACS model is a population‐based care model based on the general practice (GP) registered list that brings together a group of providers taking responsibility for delivering a full range of primary, community, mental health and hospital services for their local population (Naylor & Charles, 2018; NHS, 2016a). Its aim is to join up services to allow better decision making and more sustainable use of resources with a greater focus on prevention and integrated community‐based care and less reliance on hospital care (NHS, 2016a). At the beginning of the program, PACS had some freedom in setting their own specific performance objectives within the wider objective of joining up general practice, hospital, community and mental health services to improve population health and well‐being. Table A1 shows the objectives of the PACS and it can be seen that preventing unnecessary hospital care was a recurrent theme. Halfway through the program, receipt of additional funding was made contingent on reducing emergency admissions and length of stay in hospital (Checkland et al., 2021).

NHS England directly invested £329m in the 50 Vanguards from 2015/16–2017/18 of which £103m was used on the PACS interventions alone (NAO, 2018). The expected annual savings from 2020 to 21 were £324m. National funding for Vanguards ended in March 2018 and some of the core components of the PACS model are now being widely introduced across England (Naylor & Charles, 2018). The analysis presented here evaluates the nine PACS launched in July 2015. In each case, the PACS partners include at least one county council, one acute hospital trust and one clinical commissioning group (CCG) (NHS, 2016b). More details on the objectives, expected benefits and partners of each PACS are summarized in Table A1 in the Appendix.

## 3 DATA AND METHODS

### 3.1 Outpatient referrals and unplanned hospital admissions

We use outpatient Hospital Episode Statistics (HES) data between April 2013 and July 2018 to identify quarterly outpatient referral flows from GP practices located in the 10 English NHS CCGs of the nine PACS and their respective comparison areas. We focus exclusively on first referrals amongst individuals aged 65 years or older. We use the *Similar* 10 CCG *Explorer* tool from NHS Rightcare Intelligence to define the comparison areas (NHS, 2020). For each CCG in England, this tool identifies the 10 most similar CCGs in the country based on each CCG's demographic profile. For each PACS, we used two criteria to select the comparison group from its 10 most similar CCGs: (1) they should not be a partner of any of the 50 Vanguards; and (2) they should not be a neighbor of any CCG that is a vanguard partner. This decision was made to prevent potential spillovers from other intervention sites. For eight PACS, we found at least one CCG that met these criteria. In one case (Wirral PACS), none of the comparison candidates met the two criteria; however, one of the candidates (St Helens CCG) was a partner of an acute care collaboration vanguard that aimed to develop a network for women and children services (including maternity, gynecology, neonatal and pediatric services). Assuming this acute collaboration had a negligible effect on the care provided to patients aged 65 and older (the focus of our study), we selected St Helens CCG as the comparison group for the Wirral PACS. We then used the NHS Digital Patients Registered at a GP Practice dataset to identify GP practices in 10 intervention and 10 comparison CCGs (NHS Digital, 2019c). Table A1 in the online appendix shows the selected comparison groups for each PACS. GP practices operating within each of the intervened CCGs were assumed to be exposed homogenously to their respective PACS.

We also use HES admitted patient care data to identify unplanned hospital care measured using emergency admissions for chronic ACSAs amongst adults aged 65 or older registered in any of the GPs studied (NHS Digital, 2019b). Chronic ACSAs are those hospitalisations that might be prevented by effective care and case management. We identify them using the International Classification of Disease (ICD ‐10) codes of their primary diagnosis and include some infections, diseases of the blood, neurological disorders, mental and behavioral disorders as well as cardiovascular and respiratory diseases (NHS Digital, 2019e). An ethics approval was not required.

### 3.2 Practice and population characteristics

We merged the HES data with a number of additional variables publicly available from NHS Digital, namely, the age and sex composition of the population registered in each GP and the health care workforce available in these (NHS Digital, 2019a; NHS Digital, 2019c). Hypertension, Diabetes, Heart Failure and Obesity prevalence rates were taken from the Quality Outcome Framework indicators (NHS Digital, 2019d).

Data on registered population (list size), disease prevalence rates and workforce were not available for all the observed GPs and, thus, the GPs with missing information are excluded from the analysis. Out of the remaining 764 GPs, we identified that 91 practices (44 in intervention and 47 in comparison CCGs) closed or ceased operations during the study period 2013/14–2017/18. To the best of our knowledge, there is not a publicly available catalog of GP mergers and acquisitions in the NHS; however, using the encrypted patient identifier and the GP code where each patient is registered (both available in the inpatient HES data) we were able to identify the GPs that most likely absorbed the patients originally registered at closing GPs. This was done in four steps using both primary and secondary care data. First, we identified patients registered in closing GPs that were admitted in hospital both before and after the closure of their original GP. Second, we identified the GP code recorded for these patients in their admissions after the closure of their original GP. Third, we examined the flow of patients from closing GPs to other GPs and classified as potential absorbers those GPs recorded as the new primary care providers of more than 60% of the patients previously registered in closing GPs that were admitted to hospital both before and after the closure of their original GPs. We then compared the GP list sizes of closing GPs with their potential absorbers before the GP closure and observed if the list size of the potential absorber after the closure suggests that the potential absorber is indeed the new primary care provider of the patients originally registered in closing GPs. This process allowed us to identify the GPs that most likely absorbed patients from 81 closing GPs. It was not possible to identify a clear absorber for the remaining 10 GPs and, thus, we decided to drop them from the study. Table A2 in the online appendix shows the closing GP and its identified absorber. Absorber GPs and their absorbed practices were treated as single GPs throughout the study. This means that their list sizes, hospital admission rates and other characteristics were aggregated. The new prevalence rates of chronic diseases were computed as a weighted average based on the original list size of each GP forming the new aggregated GP. Finally, we excluded four GPs specialized in care for older and homeless people and one GP with less than 1000 registered population.

### 3.3 Measuring clinical integration

The notion of integration provides a useful way of thinking about a range of approaches that are deployed to increase coordination, cooperation, continuity, collaboration and networking across different components of health service delivery (Nolte & Mckee, 2008). Hence, integrated care seeks to improve outcomes for those with chronic health problems and complex needs by overcoming issues of fragmentation through linkage or coordination of services from different providers along the continuum of care (Nolte & Pitchforth, 2014). In this study, we focus on the clinical integration of primary and secondary care, encouraged by PACS, and we propose that one aspect hereof can be measured by using the concentration of each GP's referrals of patients to outpatient hospital care.

Referral for outpatient hospital care is one of the main points of contact between primary and secondary care that is initiated by the primary care provider, rather than, for example, patient‐initiated attendances to emergency departments. This activity therefore represents an opportunity to understand the changing relationships between hospitals and their local general practices. Referrals to specialist care have three functions: short‐term consultation for diagnosis or management, referral for long‐term management of specific illnesses, and recurrent consultation for periodic management (Starfield et al., 2005). In England, each year, more than 10M outpatient referrals are made and they represent the interface between primary and secondary care. The outpatient referral process is initiated by GPs who actively manage and maintain relationships with the specialists whom their patients see (Barnett et al., 2011). Thus, if primary care providers' work starts to integrate care more closely, we expect to be able to see a greater concentration in the number of hospitals to which they are referring patients. This is in line with the literature on referral networks which has demonstrated that referral patterns can be used to identify professional relationships between physicians (Barnett et al., 2011; Landon et al., 2012, 2013).

To build the proposed integration measure we use HES to identify all outpatient presentations by adult patients aged 65 years or older and registered in intervened and comparison practices to acute hospital trusts per quarter during 2013/14–2017/18. Then, for each GP, we compute a Herfindahl‐Hirschman Index (HHI):

(1) $$\mathrm{HHI}=\sum_{j=1}^{N}s_{ij}^{2}$$

where $s_{ij}$ is the proportion of patients referred from GP practice *i* to Hospital *j* and N is the total number of hospitals referred to by the GP practice *i*. The HHI is a measure of concentration which in our definition means that if a given GP increases the proportion of its referrals to a specific hospital, its HHI will increase as the hospital's share of the total patients that are referred to outpatient care from the GP in question is increasing (Rhoades, 1995). We interpret this concentration index as a measure of clinical integration of primary and secondary care as it captures information of the level of interaction between GPs and acute hospitals: a higher share of the referrals of a GP might reflect better coordination and exchange of information between hospitals and GPs. This measure thus relates to the “Transition facilitation” “Information transfer” and “Communication” domains of the Schultz et al. framework of care coordination measures (Schultz et al., 2013).

### 3.4 Mediation analysis

While many previous assessments of integrated care focus directly on establishing the impact of integrated care on specific outcomes, in this paper, we are interested in the causal mechanisms that can explain why integrated care may or may not produce desired outcomes. The identification of a causal mechanism requires the specification of an intermediate variable or a mediator that lies on the causal pathway between the treatment and outcome variables (Imai et al., 2011). Hence, the investigation of causal mechanisms is based on the estimation of causal mediation effects. An earlier review showed that mediation studies are generally of two types (MacKinnon et al., 2007). One type consists of investigating how a particular effect occurs, usually after finding evidence of a causal relationship between two variables. In this framework, a mediator variable is added to the analysis to improve the understanding of the relation or to determine if the relation is spurious. The second type of these studies focuses on the mediational processes; in this type of research, an intervention is designed to change mediating variables that are hypothesized to be causally related to a dependent variable. If the hypothesized relations are correct, a policy that substantially changes the mediating variables will in turn change the outcome (MacKinnon et al., 2007). The present paper represents the second type of mediation analysis.

**FIGURE 1:**
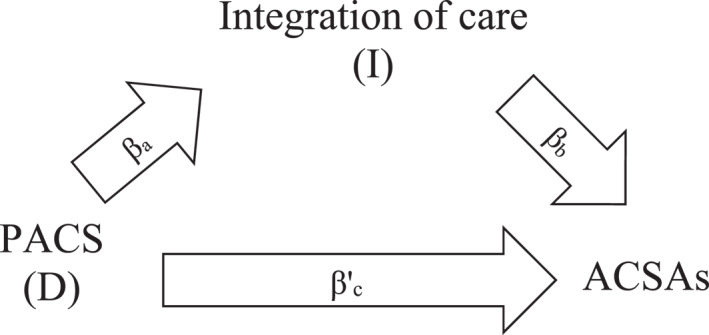
Single mediator model.

The rationale of the PACS integrated care model is that integrating health care services will allow better decision‐making and more sustainable use of resources with a greater focus on prevention and less reliance on emergency or unplanned hospital care (NHS, 2016a). We illustrate this hypothesized causal mechanism in Figure 1 as a single mediator model in which a PACS causally affects unplanned hospital care through the integration of care and other ways.

The goal of this approach is to decompose the causal effect of a treatment into the indirect effect, which represents the hypothesized causal mechanism (the combination of arrow *β*_*a*_ and arrow *β*_*b*_), and the so‐called direct effect, which represents all the other mechanisms (arrow *β*′_*c*_).

Following Anselmi et al. (2017) that applied a linear structural equation model within a difference‐in‐differences (DiD) analysis, our approach to assess mediation comprises three steps (Anselmi et al., 2017; Baron & Kenny, 1986; MacKinnon et al., 2007).

First, we estimate the impact of the PACS on integration of care using the following difference‐in differences (DiD) regression model

(2) $$I_{it}=\alpha_{i}^{1}+\gamma_{t}^{1}+\beta_{a}D_{it}+\varepsilon_{it}^{1}$$

where $I_{it}$ is the integration of care measure (HHI) of GP *i* in quarter *t* (for *t* = 1,2,…, 20). $\alpha_{i}^{1}$ and $\gamma_{t}^{1}$ are GP and quarter fixed effects, respectively. $D_{it}$ is a dummy variable taking the value of 1 if the PACS was active in GP *i* in the post‐intervention period (July 2015 ‐March 2018, quarters 11–20) and 0 otherwise, and $\varepsilon_{it}^{1}$ is the residual. The effect of the PACS on integration of care is captured by the estimation of $\beta_{a}$. If PACS have indeed contributed to a higher integration of care in their local health systems, we would expect a positive and statistically significant $\beta_{a}$ (i.e., outpatient referrals from GPs would concentrate in fewer hospitals). Model (2) is first estimated by aggregating all GP practices in the intervention and comparison groups from the nine PACS and clustering at the PACS level with bootstrapped standard errors (Cameron et al., 2008). Additionally, Equation (2) is estimated for each PACS separately. It is important to note that while an increase in the integration measure suggested here reflects a higher concentration of outpatient referrals, this concentration might not necessarily be directed toward the hospital trust participating in the PACS. Therefore, we separately confirm that any increase in the share of patients from participating GPs is directed to the hospital participating in the PACS. The reason why we cannot a priori focus only on concentration toward the PACS collaborator hospital is that GPs from control areas do not have an equivalent “key‐collaborator” hospital, which is required for our DiD setup.

In the second step, we estimate the impact of PACS on ACSAs using a DiD model

(3) $$Y_{it}=\alpha_{i}^{2}+\gamma_{t}^{2}+\beta_{c}D_{it}+\varepsilon_{it}^{2}$$

Where $Y_{it}$ is the ACSAs per 1000 adults aged 65 or older registered in GP I in quarter *t.* If PACS have indeed been successful in reducing unplanned and preventable hospital care, we would expect a negative and statistically significant $\beta_{c}$ (i.e., PACS have reduced ACSAs). Equation (3) is estimated first using all observations and then analyzing each PACS separately.

Finally, we identify the direct and indirect causal effects by adding $I_{it}$ as covariate in Equation (4):

(4) $$Y_{it}=\alpha_{i}^{3}+\gamma_{t}^{3}+\beta_{c}^{\prime }D_{it}+\beta_{b}I_{it}+\varepsilon_{it}^{3}$$

If $\beta_{b}$ is statistically significant and | $\beta_{c}^{\prime }$ | < |$\beta_{c}$ | we can infer that the effect of PACS ($D_{it}$) on ACSAs $\left(Y_{it}\right)$ is mediated through integrated care $I_{it}$ as hypothesized. $\beta_{c}^{\prime }$ measures the direct effect of $D_{it}$ on $Y_{it}$. By the standard “product method” (Baron & Kenny, 1986), the indirect or mediated effect is calculated as the product between $\beta_{a}$ and $\beta_{b}$. We use bootstrapping with 1000 replications to compute standard errors, their respective 95% confidence intervals and to assess the statistical significance of the indirect effect (Anselmi et al., 2017; MacKinnon et al., 2007). Like Equations (2) and (3), Equation (4) is estimated for the aggregated case and separately for each of the nine PACS. All models are estimated using STATA/MP 16.1.

In sum, the following conditions are required to support the causal mechanism hypothesis depicted in Figure 1

1. Significantly positive $\beta_{a}$ in Equation (2), suggesting that PACS has increased integration of care.
2. A significantly negative $\beta_{b}$ in Equation (4), that is, the higher the integration the lower the ACSA rate.
3. A significantly negative indirect effect of the policy ($\beta_{a}*\beta_{b}$)
4. A negative direct effect, but smaller in absolute terms than the total effect.

It is worth mentioning that due to the potential presence of opposing indirect and direct effects, the overall effect of the treatment (PACS) on the outcome (ACSA rate) may not be statistically significant yet mediation can still exist (MacKinnon et al., 2007).

### 3.5 Identification and specification assumptions

Imai, Keele and Tingley (2010) proposed an alternative method for estimating indirect effects by way of an algorithm that simulates predicted values of the mediator and outcome, and calculate the indirect effect on this basis. This approach is particularly useful for binary outcomes or mediators, but also has the advantage that it allows a sensitivity analysis described below. We present the results of this alternative estimation procedure (implemented using the *medeff* package by Hicks & Tingley, 2011) alongside the estimates arising from the product method. The identification of the indirect and direct effects relies on the sequential ignorability assumption (Keele, 2015). This assumption receives its name because two ignorability assumptions are made sequentially: (1) given the observed pre‐treatment confounders, the treatment assignment is assumed to be statistically independent of potential outcomes and potential mediators (i.e., ignorable) (Imai et al., 2011); and (2) The observed mediator is ignorable given the actual treatment status and pre‐treatment confounders; i.e., there are no unmeasured confounders that affect both the mediator and the outcome Imai, Keele & Tingley, 2010).

The first part of the assumption, which is identical to the assumption needed to identify the total effect, would have been satisfied if the treatment had been randomly assigned (Keele, 2015). This was not the case in PACS; however, the use of a DiD approach allows for the potential influence of unobservables affecting treatment assignment to be taken into account subject to the parallel trends assumption (Anselmi et al., 2017). We thus test whether both the pre‐intervention trends of the ACSA rate and the HHI between intervention and comparison GPs were parallel.

The second part of the sequential ignorability assumption cannot be formally tested (Imai, Keele & Tingley, 2010). However, it has been shown that when linear structural equation models are used, the sequential ignorability assumption is equivalent to $Cov\left(\varepsilon_{it}^{1},\varepsilon_{it}^{3}\right)=0$ (Imai, Keele & Tingley, 2010; Keele, 2015). That is, the residuals from the mediator model (2) and the outcome model (4) must not be correlated for the sequential ignorability assumption to hold. While this assumption is not testable, Imai et al. proposed, as a sensitivity check, calculating the correlation across the two error terms $\varepsilon_{it}^{1}$ and $\varepsilon_{it}^{3}$ at which the estimate of the indirect effect would be 0, denoted ρ.The structural equation models approach also requires that models are linear and additive (Keele, 2015). This can be tested by including a mediator‐treatment interaction in model (4) and examining if its association with the ACSA rate is significantly different from zero (Ohrnberger et al., 2020).

The assumption that PACS affect the level of integration of care which in turn affects the ACSA rate, presupposes a temporal ordering of the change in integrated care preceding that of ACSAs (Anselmi et al., 2017; MacKinnon et al., 2007). As specified in Equations (2)–(4), the suggested mediation analysis measures changes in mediators and outcomes simultaneously when, in fact, the effect of these changes may not be immediate and more time might be needed to observe significant changes caused by integrated care initiatives (Ling et al., 2012; Shaw & Levenson, 2011). For this reason, as detailed in the next section, alternative specifications that allow lags between the PACS launch and the measurement of HHI as well as between the HHI and the ACSA rate are also considered. We thus adapt models (2)–(4) to accommodate these lags by specifying

(5) $$I_{it-1}=\alpha_{i}^{4}+\gamma_{t-1}^{4}+\beta_{a}^{lag}D_{it-1}+\varepsilon_{it-1}^{4}$$

(6) $$Y_{it}=\alpha_{i}^{5}+\gamma_{t}^{5}+\beta_{c}^{lag}D_{it}+\varepsilon_{it}^{5}$$

(7) $$Y_{it}=\alpha_{i}^{6}+\gamma_{t}^{6}+{\beta^{\prime }}_{c}^{lag}D_{it}+\beta_{b}^{lag}I_{it-1}+\varepsilon_{it}^{6}$$

## 4 RESULTS

### 4.1 Testing the parallel trends assumption

We test the equivalence of linear trends between intervention and comparison GPs in the pre‐intervention period (quarters 1–9) (Ryan et al., 2015). As shown in Table A3 in the online appendix, while the parallel trends assumption for the ACSA rate was not rejected for five PACS, it was rejected for the HHI in eight of them. Furthermore, as shown in Figure A1 in the online appendix, the HHI trend suggests an anticipation effect from quarter eight in the Salford and North East Hampshire PACS.^1^ For these reasons, for each PACS separately, we matched intervention and comparison GPs on both the HHI and ACSA rate trends observed through quarters 1–8 using different matching algorithms with replacement, such as nearest neighbor, kernel and Mahalanobis (Courtemanche & Zapata, 2014; Ryan et al., 2015). The preferred matching algorithm was Mahalanobis within a propensity score caliper of 0.25 as it reached the best balance between the number of intervened GPs matched (or in the common support) and achieved reduction in the standardised bias (Stuart, 2010). Table A4 reports the matching results from the preferred matching algorithm. Table A5 in the online appendix shows that the parallel trends assumption is not rejected for any PACS in the matched sample.

**FIGURE 2:**
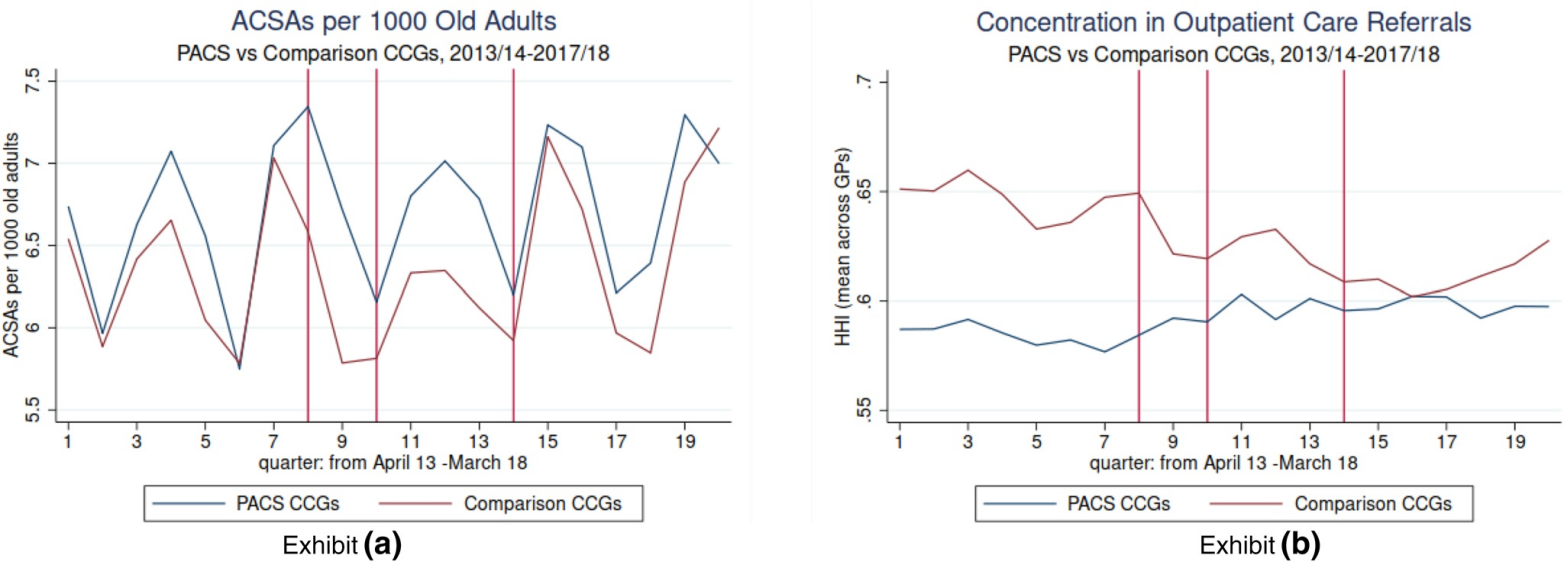
Ambulatory care sensitive emergency admission (ACSA) rate and Herfindahl‐Hirschman Index (HHI) aggregated trends. Exhibit (a). Exhibit (b). Lines in the graphs show the quarter when an anticipation behavior in some primary and acute care systems (PACS) were identified (q8), when the policy was launched (q10) and the end of the Grace period of 1 year considered in some specifications (q14).

The analysis is thus based on data from a balanced panel of 250 intervention GPs and their respective matched comparison GPs. Table 1 shows no important differences during the pre‐intervention period between the intervention and comparison GPs. It can be seen in Table 2 that only in four PACS the concentration of outpatient referrals increased from the pre‐intervention to the post‐intervention period and, surprisingly, that it decreased in four PACS. All changes in the HHI are mirrored by changes in the proportion of outpatient referrals from intervened GPs going to participating hospital trusts, thus suggesting that the variation in the concentration of referrals reflects the variation in the PACS partner's relative importance in the provision of outpatient care. In two cases, the hospital trust that partnered a PACS was not the main provider of outpatient care to the population registered in intervened GPs (measured by their share of total outpatient referrals). It is important to note that in these cases, the referrals' share of the main provider and the PACS partner also moves in the same direction as the HHI in their respective sites: it increased in South Somerset and decreased in Northumberland –not shown, but available upon request. Figure 2 displays the aggregated trends during the study period for the ACSA rate and the HHI in intervened and comparison CCGs. Figure A2 in the online appendix shows the trends followed by these variables in each PACS separately.

**TABLE 1:** Descriptive statistics.

	Pre‐intervention (quarters 1–8)			Post‐intervention (quarters 11–20)		
	PACS	Comparison	Full sample	PACS	Comparison	Full sample
	Mean (SD)	Mean (SD)	Mean (SD)	Mean (SD)	Mean (SD)	Mean (SD)
GP practices	250	250	500	250	250	500
ACSC emergency admissions per 1000 old adults	6.99 (3.94)	6.67 (3.91)	6.83 (3.93)	7.09 (3.74)	6.93 (4.10)	7.01 (3.92)
Concentration of outpatient referrals (HHI)	0.58 (0.20)	0.65 (0.17)	0.62 (0.18)	0.60 (0.17)	0.62 (0.16)	0.61 (0.17)
Outpatient referrals per registered old adult	0.20 (0.05)	0.20 (0.06)	0.20 (0.06)	0.23 (0.05)	0.23 (0.06)	0.23 (0.06)
GP list size (registered population)	8107.3 (6117)	7731.8 (4221)	7919.5 (5257.9)	8237.1 (6081.6)	7947.4 (4306.9)	8092.2 (5271)
Proportion of old adults registered in GP	0.21 (0.05)	0.21 (0.06)	0.21 (0.06)	0.22 (0.06)	0.22 (0.06)	0.22 (0.06)
Proportion of male individuals registered in GP	0.50 (0.01)	0.50 (0.02)	0.50 (0.01)	0.50 (0.01)	0.49 (0.02)	0.49 (0.02)
FTE GPs	5.40 (4.18)	5.05 (3.03)	5.22 (3.66)	4.86 (3.77)	4.35 (2.90)	4.60 (3.37)
Prevalence hypertension	15.86 (2.88)	16.28 (3.78)	16.07 (3.37)	16.16 (2.83)	16.50 (3.62)	16.33 (3.25)
Prevalence diabetes	5.92 (1.22)	6.07 (1.61)	6.01 (1.43)	6.98 (1.18)	7.19 (1.54)	7.08 (1.37)
Prevalence heart failure	0.88 (0.32)	0.89 (0.38)	0.89 (0.35)	1.01 (0.39)	0.92 (0.41)	0.97 (0.40)
Prevalence obesity	9.29 (3.21)	9.60 (3.27)	9.45 (3.24)	10.90 (3.67)	11.09 (3.44)	11.00 (3.56)
GP practice weight (times a non‐PACS GP was used as match)	1	2.72 (1.84)	1.86 (1.56)	1	2.72 (1.84)	1.86 (1.56)

Abbreviations: ACSC, Ambulatory Care Sensitive Conditions; FTE, Full Time Equivalent.

**TABLE 2:** Integration of care in intervened sites.

	Matched GP practices	PACS partner is main outpatient care provider?	Pre‐intervention		Post‐intervention	
			PACS partner's share of outpatient referrals	HHI	PACS partner's share of outpatient referrals	HHI
			Mean (SD)	Mean (SD)	Mean (SD)	Mean (SD)
Wirral	30	YES	0.64 (0.07)	0.44 (0.07)	0.67 (0.06)	0.47 (0.07)
Mid Nottinghamshire	36	YES	0.91 (0.16)	0.64 (0.14)	0.92 (0.08)	0.64 (0.10)
South Somerset	58	NO	0.15 (0.25)	0.58 (0.18)	0.17 (0.28)	0.63 (0.19)
Northumberland	35	NO	0.45 (0.10)	0.50 (0.07)	0.43 (0.09)	0.45 (0.07)
Salford	28	YES	0.47 (0.10)	0.31 (0.06)	0.63 (0.10)	0.45 (0.08)
Morecambe	27	YES	0.92 (0.07)	0.84 (0.10)	0.90 (0.06)	0.81 (0.10)
North East Hampshire	9	YES	0.82 (0.04)	0.68 (0.06)	0.86 (0.03)	0.75 (0.05)
Harrogate	16	YES	0.85 (0.13)	0.76 (0.13)	0.83 (0.12)	0.72 (0.13)
Wight	11	YES	0.90 (0.02)	0.81 (0.04)	0.78 (0.06)	0.64 (0.09)

### 4.2 Mediation analysis

Table 3 reports the results of estimating Equations (2)–(4). In the aggregated analysis, we find evidence that, overall, the PACS increased the clinical integration of care ($\beta_{a}$) which is in line with our expectations from the hypothesized causal framework. This increase is equivalent to a 7% increase with respect to the average concentration observed in intervention GPs in the pre‐intervention period. However, we do not find evidence to support the hypothesis that clinical integration of primary and secondary care is significantly linked to decreases in the ACSA rate ($\beta_{b}$) and, thus, that PACS had an impact on the ACSA rate through this type of integration. Furthermore, the overall effect of PACS on the ACSA rate ($\beta_{c}$) was not statistically although the negative sign is what we hypothesized. As shown in Table A6 these results are robust to the use of the unmatched sample.

**TABLE 3:** Mediation analysis excluding anticipation period: mediated effect of primary and acute care systems (PACS) on ambulatory care sensitive emergency admissions (ACSAs) through integrated care.

PACS	Obs	PACS effect on HHI (*β*_a_)	Relation between HHI and ACSA (*β*_b_)	Indirect effect (*β*_a_ * *β*_b_)	Indirect effect (Imai et al.)	Direct effect ($\beta_{\text{c}}^{\prime }$)	Treatment effect (*β*_c_)	Total effect [(*β*_a_ * *β*_b_) + $\beta_{\text{c}}^{\prime }$)]	ρ at which ID = 0
Aggregated PACS^a^	9000	0.04 [0.01, 0.08]**	1.80 [−0.56, 4.16]	0.08 [−0.06, 0.22]	0.08 [0.03, 0.13]***	−0.24 [−1.04, 0.56]	−0.16 [−0.95, 0.63]	−0.16 [−0.95, 0.63]	0.04
Wirral	1080	0.04 [0.03, 0.06]***	0.47 [−3.59, 4.53]	0.02 [−0.16, 0.20]	0.03 [−0.15, 0.21]	0.23 [−0.51, 0.96]	0.25 [−0.49, 0.98]	0.25 [−0.49, 0.98]	0.01
Mid Nottinghamshire	1296	0.09 [0.08, 0.11]***	2.89 [0.11, 5.67]**	0.27 [0.01, 0.54]**	0.28 [0.03, 0.55]**	1.43 [0.72, 2.15]***	1.71 [1.03, 2.38]***	1.71 [1.03, 2.38]***	0.07
South Somerset	2088	0.08 [0.08, 0.09]***	0.29 [−1.96, 2.54]	0.02 [−0.16, 0.21]	0.03 [−0.15, 0.21]	−0.36 [−0.76, 0.03]*	−0.34 [−0.68, 0.00]**	−0.34 [−0.68, 0.00]**	0.01
Northumberland	1260	−0.01 [−0.02, 0.01]***	−1.38 [−4.91, 2.15]	0.02 [−0.03, 0.07]	0.02 [−0.03, 0.07]	0.27 [−0.28, 0.82]	0.29 [−0.27, 0.84]	0.29 [−0.27, 0.84]	−0.02
Salford	1008	0.11 [0.10, 0.12]***	5.43 [−0.81, 11.67]*	0.59 [−0.09, 1.28]*	0.59 [−0.11, 1.38]	−2.43 [−3.75, −1.11]***	−1.84 [−2.91, −0.76]***	−1.84 [−2.91, −0.76]***	0.06
Morecambe	972	0.00 [−0.01, 0.01]	5.57 [1.48, 9.67]***	0.00 [−0.06, 0.06]	−0.00 [−0.06, 0.07]	−1.90 [−2.49,−1.31]***	−1.90 [−2.49, −1.31]***	−1.90 [−2.49, −1.31]***	0.10
North East Hampshire	324	0.10 [0.08, 0.12]***	−0.06 [−7.52, 0.72]	−0.01 [−0.73, 0.72]	−0.03 [−0.78, 0.70]	0.00 [−1.31, 1.31]	−0.01 [−0.97, 0.96]	−0.01 [−0.97, 0.96]	−0.00
Harrogate	576	−0.01 [−0.02, 0.01]	0.31 [−4.82, 5.44]	0.00 [−0.05, 0.04]	−0.00 [−0.05, 0.05]	−0.15 [−0.77, 0.48]	−0.15 [−0.77, 0.48]	−0.15 [−0.77, 0.48]	0.01
Isle of Wight	396	−0.16 [−0.19, 0.13]***	−3.60 [−7.93, 0.74]	0.57 [−0.12, 1.26]	0.58 [−0.13, 1.35]	0.02 [−1.01, 1.06]	0.59 [−0.23, 1.42]	0.59 [−0.23, 1.42]	−0.11

*Note*: 95% confidence intervals in brackets (built with bootstrapped standard errors, 1000 replications).

^a^ Confidence intervals built with clustered standard errors at the PACS level and bootstrapped with 1000 replications.

*p < 0.10, **p < 0.05, ***p < 0.01.

When analyzing each PACS in separate analyses, the positive effect of PACS on clinical integration persists in five out of the nine PACS with the highest increase observed in Salford. In two cases (Morecambe and Harrogate), the results suggest that PACS did not have an effect on our measure of clinical integration. In Northumberland and in the Isle of Wight, contrary to the hypothesized mechanism, the PACS had a negative effect on clinical integration of primary and secondary care. That is, following the launch of PACS, the concentration of outpatient referrals decreased in both areas.

Mid Nottinghamshire and Morecambe are the only PACS where the relation between the HHI and ACSAs ($\beta_{b}$) is statistically significant (at the 5% and 1% level, respectively) although with a sign contrary to our expectations. Our findings suggest that higher concentration is associated with higher ACSA rates. We also find weak evidence (at the 10% significance level) of a similar effect in Salford.

The overall effect of the policy on the ACSA rate is negative and significant in three cases: South Somerset, Salford and Morecambe. The effect ranges between 0.34 and 1.90 fewer ACSAs per 1000 older adults per quarter as a result of PACS. Given the lack of a significant association between HHI and the ACSA rate in South Somerset and the lack of an effect of the policy on the HHI in Morecambe, there is no evidence that the effects on the ACSA rate in these two PACS were mediated by the integration of primary and secondary care. We find weak evidence that the positive relation between HHI and the ACSA rate in Salford may have prevented a further decrease in the ACSA rate to the one observed. In other words, the indirect and the direct effects of PACS on the ACSA rate might have worked in different directions, but the negative direct effect more than offset the positive indirect effect.

Contrary to this result, the overall effect of PACS on the ACSA rate in Mid Nottinghamshire is positive and significant at the 1% level. We found that there were 1.71 more ACSAs per 1000 older population and that this effect was mediated by the integration of primary and secondary care. That is, the increase in the concentration of outpatient referrals contributed with 16% of the increase in the ACSA rate of Mid Nottinghamshire.

To account for variables that could potentially be related to treatment assignment, to the integration of care and/or to the ACSA rate, we include a vector of GP characteristics $X_{it}$ in models (2)–(4) and report the results of these alternative specifications in the online appendix (Table A7). Following Exley et al. (2019), the covariates incorporated in $X_{it}$ are the total GP list size, the proportion of patients registered with the practice aged 65 and over, the proportion of patients that were male and the number of Full Time Equivalent GPs in the practice (Exley et al., 2019). $X_{it}$ also includes the prevalence rates of hypertension, diabetes, heart failure and obesity amongst the registered GP population. In general, our results are robust to the inclusion of these covariates.

Table 4 presents the results of the analysis that acknowledges the temporal ordering between the integration of care and the ACSA rate by allowing one‐quarter lag between the measurement of the HHI and the ACSA rate (Equations 5–7). The results of the aggregated analysis are robust to those estimated with the model measuring HHI and the ACSA rate simultaneously. Likewise, the signs, magnitudes and significance of the impact of each PACS on the integration measure are similar to those reported in Table 3. Three differences between the analyses presented in Tables 3 and 4 are worth noting. First, while there is an overlap in the 95% confidence intervals of results in both analyses, the contribution of the mediated effect on the total PACS effect in Mid Nottinghamshire increased from 16% to 27%. Second, the association between integration and the ACSA rate observed in Salford is no longer statistically significant and, thus, the suggestion that this relationship prevented a larger decrease on the ACSA rate does not hold under this alternative specification. Third, for the Harrogate PACS, $\beta_{b}$ in the lagged specification is now significant at the 1% and much higher than in the simultaneous analysis although the null effect of the policy on integration of care prevails.

**TABLE 4:** Mediation analysis excluding anticipation period with one quarter lag between Herfindahl‐Hirschman Index (HHI) and ambulatory care sensitive emergency admission (ACSA) rate.

PACS	Obs	PACS effect on HHI (*β*_a_)	Relation between HHI and ACSA (*β*_b_)	Indirect effect (*β*_a_ * *β*_b_)	Indirect effect (Imai et al.)	Direct effect ($\beta_{\text{c}}^{\prime }$)	Treatment effect (*β*_c_)	Total effect [(*β*_a_ * *β*_b_) + $\beta_{\text{c}}^{\prime }$)]	ρ where ID = 0
Aggregated PACS^a^	8000	0.05 [0.01, 0.08]**	1.25 [−1.59, 4.09]	0.06 [−0.09, 0.20]	0.06 [0.01, 0.11]**	−0.26 [−1.03, 0.51]	−0.20 [−1.03, 0.62]	−0.20 [−1.03, 0.62]	0.03
Wirral	960	0.05 [0.03, 0.06]***	−2.13 [−6.38, 2.13]	−0.10 [−0.30, 0.10]	−0.10 [−0.32, 0.11]	0.11 [−0.65, 0.87]	0.01 [−0.74, 0.76]	0.01 [−0.74, 0.76]	−0.04
Mid Nottinghamshire	1152	0.09 [0.08, 0.11]***	5.08 [2.37, 7.79]***	0.47 [2.37, 0.73]***	0.47 [0.21, 0.76]***	1.25 [0.46, 2.04]***	1.72 [0.99, 2.45]***	1.72 [0.99, 2.45]***	0.12
South Somerset	1856	0.09 [0.08, 0.09]***	1.13 [−1.45, 3.72]	0.10 [−0.13, 0.33]	0.09 [−0.13, 0.32]	−0.49 [−0.92, −0.05]**	−0.39 [−0.76, −0.02]**	−0.39 [−0.76, −0.02]**	0.02
Northumberland	1120	−0.01 [−0.02, −0.01]***	−0.22 [−3.95, 3.51]	0.00 [−0.05, 0.06]	0.00 [−0,05, 0.06]	0.42 [−0.16, 1.00]	0.43 [−0.15, 1.01]	0.43 [−0.15, 1.01]	−0.00
Salford	896	0.11 [0.09, 0.12]***	−0.57 [−6.56, 5.41]	−0.06 [−0.71, 0.58]	−0.07 [−0.71, 0.55]	−1.67 [−3.17, −0.17]**	−1.73 [−2.94, −0.53]***	−1.73 [−2.94, −0.53]***	−0.01
Morecambe	864	0.00 [−0.01, 0.01]	−1.08 [−5.30, 3.15]	0.00 [−0.03, 0.03]	0.00 [−0.03, 0.04]	−2.26 [−2.88, −1.65]***	−2.26 [−2.88, −1.64]***	−2.26 [−2.88, −1.64]***	−0.02
North East Hampshire	288	0.09 [0.07, 0.11]***	−5.55 [−13.64, 2.55]	−0.52 [−1.27, 0.24]	−0.50 [−1.25, 0.23]	0.40 [−0.92, 1.71]	−0.12 [−1.18, 0.94]	−0.12 [−1.18, 0.94]	−0.10
Harrogate	512	0.00 [−0.02, 0.01]	13.93 [9.52, 18.34]***	−0.05 [−0.23, 0.12]	−0.06 [−0.21, 0.10]	0.00 [−0.62, 0.62]	−0.06 [−0.69, 0.58]	−0.06 [−0.69, 0.58]	0.25
Isle of Wight	352	−0.15 [−0.18, −0.12]***	−1.67 [−6.04, 2.70]	0.25 [−0.42, 0.92]	0.27 [−0.40, 0.87]	0.30 [−0.85, 1.46]	0.56 [−0.30, 1.41]	0.56 [−0.30, 1.41]	−0.05

*Note*: 95% confidence intervals in brackets (built with bootstrapped standard errors, 1000 replications).

^a^ Confidence intervals built with clustered standard errors at the PACS level and bootstrapped with 1000 replications.

*p < 0.10, **p < 0.05, ***p < 0.01.

In another alternative specification, we consider a grace period of 1 year after the launch of the policy recognizing that PACS may need to mature before influencing care integration (Shaw & Levenson, 2011). This is done by excluding data from the first four quarters after the launch of PACS (quarters 10–13) from the analysis. Then, as above, we introduce a one‐quarter lag between the measurement of integration and the ACSA rate. The findings of this analysis are reported in Table 5. In general, results are robust to previous specifications. There are, however, three main differences. First, the overall negative and significant effect of PACS on the ACSA rate in South Somerset found in the previous analyses is not significant anymore. Second, we find weak evidence that the concentration of outpatient referrals is negatively associated to the ACSA rate in Morecambe, but the effect of the policy on the ACSA rate is still not different from zero. Thus, these findings still suggest no mediation effect of integration of care on ACSAs in Morecambe. Third, in North East Hampshire we find that the relationship between integration of care and the ACSA rate is negative and significant which combined with the positive effect of PACS on integration yields a negative and significant indirect effect which appears to drive a decrease in the ACSA rate as a result of the policy. This last result is only significant at the 10% level.

**TABLE 5:** Mediation analysis excluding anticipation period with 1 year of grace period and one quarter lag between Herfindahl‐Hirschman Index (HHI) and ambulatory care sensitive emergency admission (ACSA) rate.

PACS	Obs	PACS effect on HHI (*β*_a_)	Relation between HHI and ACSA (*β*_b_)	Indirect effect (*β*_a_ * *β*_b_)	Indirect effect (Imai et al.)	Direct effect ($\beta_{\text{c}}^{\prime }$)	Treatment effect (*β*_c_)	Total effect [(*β*_a_ * *β*_b_) + $\beta_{\text{c}}^{\prime }$)]	ρ where ID = 0
Aggregated PACS^a^	6500	0.05 [0.01, 0.10]**	1.32 [−1.85, 4.50]	0.07 [−0.12, 0.25]	0.07 [−0.01, 0.14]	−0.41 [−1.25, 0.44]	−0.34 [−1.24, 0.558]	−0.34 [−1.24, 0.558]	0.03
Wirral	780	0.07 [0.06, 0.09]***	−2.52 [−0.54, 2.39]	−0.18 [−0.54, 0.17]	−0.18 [−0.57, 0.18]	0.12 [−0.83, 1.08]	−0.06 [−0.97, 0.85]	−0.06 [−0.97, 0.85]	−0.04
Mid Nottinghamshire	936	0.11 [0.09, 0.13]***	4.81 [1.63, 7.98]***	0.54 [0.17, 0.92]***	0.53 [0.18, 0.93]***	1.13 [0.28, 1.97]***	1.67 [0.89, 2.45]***	1.67 [0.89, 2.45]***	0.11
South Somerset	1508	0.09 [0.08, 0.10]***	1.88 [−0.99, 4.75]	0.16 [−0.09, 0.41]	0.16 [−0.10, 0.43]	−0.38 [−0.87, 0.12]	−0.22 [−0.64, 0.21]	−0.22 [−0.64, 0.21]	0.04
Northumberland	910	−0.01 [−0.02, 0.00)**	−0.25 [−4.46, 3.96]	0.00 [−0.04, 0.05]	0.00 [−0.05, 0.06]	0.12 [−0.52, 0.77]	0.12 [−0.52, 0.77]	0.12 [−0.52, 0.77]	
Salford	728	0.12 [0.11, 0.14]***	1.64 [−4.77, 8.05]	0.20 [−0.59, 1.00]	0.20 [−0.61, 0.97]	−2.39 [−4.06, −0.72]***	−2.19 [−3.53, −0.84]***	−2.19 [−3.53, −0.84]***	0.02
Morecambe	702	0.00 [−0.01, 0.02]	−4.12 [−8.99, 0.75]*	−0.02 [−0.09, 0.05]	−0.02 [−0.09, 0.03]	−2.52 [−3.21, −1.83]***	−2.54 [−3.23, −1.85]***	−2.54 [−3.23, −1.85]***	−0.07
North East Hampshire	234	0.08 [0.06, 0.11]***	−9.16 [−17.43, −0.90]**	−0.77 [−1.49, −0.05]**	−0.78 [−1.53, −0.13]*	−0.21 [−1.66, 1.24]	−0.98 [−2.15, 0.18]*	−0.98 [−2.15, 0.18]*	−0.16
Harrogate	416	−0.01 [−0.02, 0.01]	11.47 [6.51, 16.43]***	−0.08 [−0.25, 0.09]	−0.09 [−0.27, 0.08]	−0.07 [−0.78, 0.64]	−0.15 [−0.86, 0.56]	−0.15 [−0.86, 0.56]	0.21
Isle of Wight	286	−0.22 [−0.25, −0.20]***	−4.13 [−12.57, 4.30]	0.92 [−0.95, 2.80]	0.94 [−1.02, 2.74]	−0.38 [−2.69, 1.94]	0.55 [−0.41, 1.50]	0.55 [−0.41, 1.50]	−0.10

*Note*: 95% confidence intervals in brackets (built with bootstrapped standard errors, 1000 replications).

^a^ Confidence intervals built with clustered standard errors at the PACS level and bootstrapped with 1000 replication.

*p < 0.10, **p < 0.05, ***p < 0.01.

To analyze how sensitive the results are to the lag‐length choice, in two separate specifications, we include lags of one semester and 1 year between the integration measure and the ACSA rate (see Table A8). Results are robust on the whole to the lag choice although with two important differences. Using a 6‐months lag we find a significant negative effect of the PACS on the integration of care in Northumberland and that this integration is negatively associated with the ACSA rate. Therefore, we find evidence of a positive mediated effect of integration of care on the ACSA rate that nevertheless did not translate into a significant total effect on ACSAs due to the imprecision on the estimation of the direct effect that seems to work on the opposite direction. When the 1‐year lag is used, the relation between integration of care and the ACSA rate is not significant anymore in Mid Nottinghamshire and, thus, the mediated effect of integration of care becomes statistically insignificant too.

### 4.3 Sensitivity tests

The estimates of the indirect effect using the Imai, Keele and Tingley, (2010) algorithm are very close to the estimates from the product method. Using this approach, the indirect effect in the aggregate analyses, however, becomes statistically significant at the 5% level in the analyses presented in Tables 3 and 4.

As mentioned above, the identification of the indirect and direct effects requires the sequential ignorability assumption which cannot be tested. Instead, we present estimates of the correlation coefficient ρ at which the estimated indirect effect would be 0. Considering the estimate of the indirect effect for Mid Nottinghamshire, which is consistently statistically significant, the interpretation of ρ is that, to conclude that the true indirect effect is not statistically significantly different from zero, an unobserved confounder affecting both clinical integration and ACSAs in the same direction must exist, making the correlation between the two error terms greater than 0.11. Imai, Keele, Tingley and Yamamoto (2010) caution that there is no rule of thumb to suggest what represents an “acceptable” *ρ* at which the indirect effect would be 0, but suggest that if more studies begin reporting this entity, it will be possible to compare estimates in the literature from any specific application. In our case, we are not aware of other studies using mediation analysis to the setting of integrated care, and so we have no ideal comparator available. If we instead compare to estimates from Anselmi et al. (2017) who used a similar approach, we find that they reported ρ′s at which the indirect effect would be 0 in the interval from [0.03] to [0.26], that is, similar to the estimates we report. However, we caution that this study was from a very different context (Tanzania) and concerned a different type of intervention (Pay for performance) which should be kept in mind when evaluating our sensitivity estimates. The use of structural models in this paper also assumes that models are linear and additive (Keele, 2015). We test this assumption by including a mediator‐treatment interaction in Equations (4) and (7) (see Table A9). With only one exception, there is no strong evidence that suggests a violation of the linearity assumption; therefore, these tests support the sequential ignorability assumption. The linearity assumption is rejected in the one‐quarter lag model for Wirral at the 5% significance and in the simultaneous model for North East Hampshire at the 10% significance level. We thus suggest caution in the interpretation of the results for these PACS. As shown in Table A10, the results of the aggregated analysis are robust to the exclusion of these two PACS.

## 5 DISCUSSION

Existing evaluations of integrated care have focused on the impact of programmes on health outcomes, often finding none or even negative effects on emergency and preventable hospital care (Busse & Stahl, 2014; Exley et al., 2019; Huntley et al., 2013; O'Neill et al., 2017; Stokes et al., 2015). The lack of identified effects calls for a rethinking of the focus of integrated care evaluations. Therefore, in this paper we propose instead to begin evaluations of integrated care by addressing two fundamental questions: (1) do integrated care programmes in fact lead to greater clinical integration, and (2) is greater clinical integration associated with improved outcomes? We illustrated this analytical approach by re‐examining the evaluation of the integrated PACS program which was part of the New Care Models program in the English NHS.

We first posited that one aspect of the clinical integration of primary and secondary care can be measured using the concentration of outpatient referrals from GPs. We evaluated the effect of the nine integrated PACS on integrating primary and secondary care and finally decomposed the effect of PACS on ambulatory care sensitive conditions amongst adults aged 65 or older. The evidence found in this analysis questions the hypothesized causal mechanism that PACS reduce preventable emergency hospital care through greater integration.

While we found strong evidence that the PACS have indeed increased clinical integration of care in five PACS, the relation between greater integration and ACSAs is generally non‐significantly different from zero. In one case we found evidence suggesting that a higher level of integration was associated with a higher ACSA rate. Only in two PACS and only in the models where we allowed for a long lag between the measurement of integration and the ACSA rate, we observed a significantly negative relation between these variables which did not translate into the hypothesized mediated effect of integration on PACS.

Furthermore, in the aggregated analyses we found that the direct and total effects of PACS on the ACSA rate were not different from zero. These findings remained unchanged in five of the separate analyses for each PACS. From the remaining four PACS, we found significantly negative effects in three PACS and positive effects in one. These findings are consistent with the story of mixed results on integrated care policies in England found in the recent literature (Exley et al., 2019; O'Neill et al., 2017; Roland et al., 2012; Morciano et al., 2020), but here we tested the hypothesized causal mechanism and found no evidence to support the premise that higher clinical integration of primary and secondary care leads to less unplanned hospital care. Recent research has already started to unpack the underlying processes leading to this outcome (e.g., Checkland et al., 2021). Our findings may motivate further mixed‐methods research to explore why the relationship between unplanned hospital care and integration of primary and secondary care is generally different to the one hypothesized, and to understand why the ACSA rate has decreased in areas where integration of care, as measured here, has not played a mediator role after the PACS implementation.

To become a vanguard prototype, sites had to submit expressions of interest. Only 50 of 380 submitted applications were successful (NAO, 2018). Hence, unobservable factors that may influence both the likelihood of treatment assignment and policy success could exist, for example, collaborations formed amongst the PACS partners before the launch of the program. If the matching algorithm used to define the comparison groups, based on the historic trends of both the mediator and the outcome variables, did not fully account for unobserved confounders affecting the likelihood of becoming a PACS this could bias our results. However, we did not find strong evidence suggesting unobserved confounding between the treatment and the mediator as the interaction of these variables was not significantly different from zero in the ACSA rate model. An alternative to account for unobserved confounding followed in recent studies uses instrumental variables (IV) within the mediation analysis framework (Huber, 2019; Ohrnberger et al., 2020). While this method allows for the possibility of unobserved confounding between the mediator and the outcome, the exclusion restriction –crucial in IV– implies assuming that the direct effect of the policy is zero (Keele, 2015). That is, the use of IV in mediation analysis assumes that the effect of the treatment is entirely mediated. This does not seem to be a credible assumption in the PACS case as integration is a multidimensional concept and we cannot rule out the possibility that in some PACS other ways of integration not captured with the concentration of outpatient referrals are indeed playing an important role in the variation of the ACSA rate.

There are limitations of our work that should be mentioned. First, measuring the clinical integration of care with the HHI of concentration may be subject to an endogeneity problem caused by either GPs and/or patients choosing to refer (or being referred) to hospital trusts providing better outpatient quality. The unplanned admissions rate at the GP level can be interpreted as an indicator of the quality provided by the GP in question, thus there could be a problem of reverse causality if GPs providing better quality of care (lower unplanned admissions rate) tend to be better integrated with hospital trusts providing better outpatient care. These problems are usually addressed in the literature by using predicted patient flows rather than observed flows to compute the HHI (Moscelli et al., 2016, 2019). Since our interest is to assess the effect of PACS on integration of care and to use the latter to estimate a mediation effect on unplanned admissions, by using predicted flows instead of actual patient flows we will be missing the variation we are interested in capturing. In our case, this type of endogeneity could be problematic if a policy encourages GPs to integrate with a hospital trust perceived as providing lower quality of outpatient care (in comparison with neighboring trusts) as GPs may choose not to integrate. The results presented here suggest that the policy had an effect on the integration measure and thus does not indicate that our analysis is affected by this type of endogeneity.

We also acknowledge that the choice of ACSAs as our outcome of interest only captures one of the multiple possible effects PACS could have had. It is possible that PACS generated improvements in other dimensions of health and wellbeing which our analysis does not assess. Future research wishing to unpack the effects of integrated care using a mediation approach should aim to do so using outcome measures that capture the intended impact of programmes as fully as possible.

Thirdly, to acknowledge the temporal ordering between the integration of care and the ACSA rate we introduced a lag between the measurement of the concentration of outpatient referrals and the ACSA rate. Thus, the implicit assumption is that integration of care only affects the future ACSA rate but ignores any simultaneous effect. We believe though that this is a feasible assumption given that the ACSAs considered here are typically a reflection of suboptimal management of chronic conditions and not necessarily the immediate consequences of the quality of the primary care delivered. Recent developments in the biostatistics literature, account for the cumulative effect of time varying mediators on the outcome by using marginal structural models and inverse probability weighting (Vanderweele & Tchetgen Tchetgen, 2017). However, the construction of the weights requires the estimation of a series of models that become very easily overidentified when, as in our case, there is a small number of treated units. Moreover, this method is more sensitive than a DiD approach to differences in the levels of the outcome and mediator variables at baseline between intervened and comparison units which complicates its use in the evaluation of non‐randomised policies.

Additionally, the definition of intervention and comparison groups may have biased the estimated treatment effects downwards in two ways. First, the assumption that all GPs operating within an area of a PACS were exposed homogenously to their respective PACS ignores the implementation barriers faced by each of the nine PACS. Previous studies have suggested that the implementation of PACS may not have been homogeneous within and across intervention sites (NAO, 2018; Starling, 2017). We addressed the heterogeneity across sites by conducting separate analyses for each PACS to identify differential effects of the policy across intervention sites. However, as no catalog of GPs actively participating in PACS exists, we cannot rule out the possibility that some of the GPs that were considered here as intervention practices did not react to the policy. In that case, our estimates are a lower‐bound of the PACS effects on both the HHI and the ACSA rate. The estimated treatment effects presented here may also be underestimated as the selected comparison groups for the South Somerset and Salford PACS (Kernow CCG and Stoke‐on‐Trent CCG, respectively) were previously part of the Integrated Care Pioneers which was a program that promoted integration between local health and social care systems in England and that preceded the NHS New Care Models. The fact that these two comparison sites were exposed to a previous policy could also mean that the estimated effects for the respective PACS may be underestimated. However, despite this potential underestimation, the results for both the South Somerset and Salford PACS are generally consistent with the findings for the rest of PACS whose comparison groups were not exposed to similar national integrated care policies.

Finally, by focusing only on the interactions between GPs and clinical specialists in the outpatient setting, we capture just one out of several potential forms of coordination amongst health care providers that are encouraged by PACS. These include, for example, the integration of specialist doctors and nurses into neighborhood care teams. Our findings are thus not necessarily representative of all forms of integration that might occur in the local health system as a consequence of PACS, but arguably an important one. Future research might focus on using other data sources to incorporate additional relationships between health and social care providers into other measure of integrated care for application in the framework we suggest.

## CONFLICT OF INTEREST STATEMENT

Dr. Kristensen reports grants from UK Medical Research Council, non‐financial support from Imperial NIHR Patient Safety Research Center, during the conduct of the study. While undertaking the analysis presented in the submitted work, Dr Lugo‐Palacios was employed by Imperial College London. His salary was paid from a UK Medical Research Council grant of which Dr Søren Rud Kristensen was the Principal Investigator. Dr. Clarke reports grants from Wellcome Trust, during the conduct of the study.

## Supporting information

Supplementary Material

## Data Availability

Data on the age and sex composition of the population registered in each GP, practice workforce and practice level Hypertension, Diabetes, Heart Failure and Obesity prevalence rates are freely available from the website of NHS Digital (https://digital.nhs.uk/data). Data on Ambulatory Care Sensitive Admission rates were based on Hospital Episodes Statistics which require permission to access. Details on the application process can be found here: https://digital.nhs.uk/services/data‐access‐request‐service‐dars.

## ENDNOTE

^1^
The first 29 Vanguards including the PACS were chosen in March 2015. We take July 2015 as the start of the policy as it was when the original support package was published. This lag may explain an anticipation effect in the integration of primary and secondary care.
