# Supplementary material for: Back to basics: A mediation analysis approach to addressing the fundamental questions of integrated care evaluations
Source: Health Econ. 2023 May 26;32(9):2080–97. doi: 10.1002/hec.4713 (PMC10947178; doi:10.1002/hec.4713)

**Table A1.** PACS objectives, partners and comparison areas

| **Vanguard** | **Objectives^1^** | **Actions planned** | **Expected benefits** | **Intervened CCGs** | **Vanguard partners** | **Population** | **Comparison CCGs^2^** |
| --- | --- | --- | --- | --- | --- | --- | --- |
| South Somerset Symphony Programme | To develop new ways of caring for patients that either help to prevent ill health or address any potential problems as early as possible | GPs, hospital clinicians, therapists, social workers and patients will work together to develop packages of care which cover all of an individual's care and lifestyle needs | Partners will help prevent ill health and reduce reliance on health and care services | NHS Somerset CCG | Yeovill District Hospital NHS Foundation Trust | 200.000 | NHS Kernow CCG |
|  |  |  |  |  | Somerset GP Federation |  |  |
|  |  |  |  |  | Somerset CCG |  |  |
|  | Services will be available closer to where people live and patients will be supported to stay independent and healthier for longer and avoid unecessary hospital admissions |  | Patients will receive care earlier and quicker thanks to a more efficient use of resources |  | Somerset County Council |  |  |
|  |  |  |  |  |  |  |  |
|  |  |  |  |  |  |  |  |
|  |  |  |  |  |  |  |  |
| Northumberland Accountable Care Organisation | To help teams across different organisations to work more effectively together, with the same shared goals | Develop a single accountable care organisation: overarching organisation that sits above a joined up health and social care system made up of a number of different providers | Improved outcomes for seriously ill patients requiring emergency hospital care | NHS Northumberland CCG | Northumbria Healthcare NHS Foundation Trust | 320.000 | NHS East Riding of Yorkshire CCG |
|  |  |  |  |  | NHS Northumberland CCG |  |  |
|  |  |  |  |  | Northumberland County Council |  |  |
|  | To improve access to GP advice and explore the potential for new networks of GP practices to work together to extend access |  | Reduced reliace on emergency care and hospital admissions |  |  |  |  |
|  |  | Multi-disciplinary (GPs, specialists, pharmacists and community nursing teams)teams will work together to look after patients who are most vulnerable |  |  |  |  |  |
|  |  |  | Support the future efficiency and financial stability of the health and social care system as a whole |  |  |  |  |
|  |  |  |  |  |  |  |  |
|  |  |  |  |  |  |  |  |
|  |  |  |  |  |  |  |  |
| Salford Together | To improve health and social care for older people through better links between GPs, district nurses, social workers, mental health professionals, care homes, voluntary organisations and local hospitals | New focus on preventing ill health through increased screening for health conditions | Create more effective and efficient personalised services | NHS Salford CCG | Salford City Council | 230.000 | NHS Stoke on Trent CCG |
|  |  |  |  |  | NHS Salford CCG |  |  |
|  |  |  |  |  | Salford Royal NHS Foundation Trust |  |  |
|  |  | New contact centre supporting the multi-disciplinary groups of staff and coordinating the use of telecare | Patients will be kept safely at home for longer and receive care closer to home instead of in hospital |  | Greater Manchester West Mental Health Trust |  |  |
|  | Better communication and planning between professionals and services involved in an individual's care to deliver high quality of care effectively and efficiently |  |  |  |  |  |  |
|  |  |  |  |  |  |  |  |
|  |  |  |  |  |  |  |  |
| Wirral Partners | To provide the environment for the diverse workforce to deliver outstanding care and support to the population | Commissioning services jointly, supporting joined-up local services, and working in partnership with the local communities and voluntary sector | Improving health and wellbeing outcomes | NHS Wirral CCG | NHS Wirral CCG | 330.000 | NHS St Helens CCG |
|  |  |  |  |  | Wirral Council |  |  |
|  |  |  | Improving patients' and service users' experience |  | Wirral University Teaching Hospital NHS Foundation Trust |  |  |
|  |  | Developing a new system where electronic patient records are shared across all health providers to better plan the services that the population needs and imporve the efficiency of the health and care system |  |  | Wirral Community NHS Trust |  |  |
|  |  |  | Providing efficient, well organised and value for money health and social care services |  | Cheshire and Whirral Partnership NHS Foundation Trust |  |  |
|  |  |  |  |  |  |  |  |
|  |  |  |  |  |  |  |  |
|  |  |  |  |  |  |  |  |
| Mid Nottinghamshire Better Together | Getting doctors, nurses, other health professionals and social care staff to work more closely together to support the needs of patients and carers | Focus on a) urgent and proactive care; and b) early and planned care | Reduce pressure on hospitals by helping patients leave hospital sooner | 1) NHS Mansfield and Ashfield CCG | Nottingham Emergency Medical Services | 310.000 | NHS North East Lincolnshire CCG |
|  |  |  |  |  | East Midlands Ambulance Service NHS Trust |  |  |
|  |  | Creation of new integrated care teams (including GPs, specialist nurses, social workers and a voluntary sector worker) to provide better, joined-up care for patients who are at risk of being admitted to hospital |  |  | Nottinghamshire County Council |  |  |
|  |  |  | Patients receive more and better care closer to home |  | Nottinghamshire Healthcare NHS Foundation Trust |  |  |
|  | More co-ordinated care for patients and better working relationships and clearer processes for staff |  |  |  | Nottingham University Hospitals NHS Trust |  |  |
|  |  |  |  | 2) NHS Newark and Sherwood CCG | Sherwood Forest Hospitals NHS Foundation Trust |  | NHS North Lincolnshire CCG |
|  |  |  |  |  | United Lincolnshire Hospitals NHS Trust |  |  |
|  |  | New process (transfer of care) allowing patients to be discharged from hospital as soon as they are medically fit |  |  | Together Everyone Achieves More |  |  |
|  |  |  |  |  |  |  |  |
| Better Care Together  (Morecambe Bay Health Community) | To deliver consistent care for individuals with priorities localised | Creation of twelve new health and care teams (nurses, doctors, social workers) from across primary, secondary and community care based in local areas. They will work together in three clinical networks | Improved consistency and quality of care, both in and out of hospital | NHS Morecambe Bay CCG  (established in April 2017 following the integration of former NHS Lancashire North CCG and the 32 GPS of the South Cumbria network of the NHS Cumbria CCG) | University Hospitals of Morecambe Bay NHS Foundation Trust | 365.000 | NHS North East Essex CCG |
|  |  |  |  |  | Cumbria Partnership NHS Foundation Trust |  |  |
|  | People going to hospital only when they need the specialist care that only a hospital can provide |  |  |  | North West Ambulance Service |  |  |
|  |  |  | Hospitals concentrating on those patients who need specialist treatment or emergency care |  | Blackpool Teaching Hospitals NHS Foundation Trust |  |  |
|  |  |  |  |  | Lancashire Care NHS Foundation Trust |  |  |
|  |  |  |  |  | Lancashire County Council |  |  |
|  |  |  | A health system with exciting and innovative teams working together |  | Cumbria County Council |  |  |
|  |  |  |  |  | NHS Lancashire North CCG |  |  |
|  |  |  |  |  | NHS Cumbria CCG |  |  |
|  |  |  |  |  | North Lancashire Medical Services |  |  |
|  |  |  |  |  | South Cumbria Primary Care Collaborative |  |  |
| Happy Healthy at Home (North East Hampshire and Farnham) | To keep patients as well as possible and out of hospital where appropriate | New integrated teams of specialist health and social care professionals who will ensure joined up care for patients, especially those who are vulnerable or have complex needs | Close the gap between the available resources and the costs of providing services to meet needs | NHS North East Hampshire and Farnham CCG | NHS North East Hampshire and Farnham CCG | 220.000 | NHS South Gloucestershire CCG |
|  |  |  |  |  | Frimley Health NHS Foundation Trust |  |  |
|  |  |  |  |  | Southern Health NHS Foundation Trust |  |  |
|  |  |  |  |  | Surrey and Borders Partnership NHS Foundation Trust |  |  |
|  |  | Teams include community nurses, occupational therapists, physiotherapists, social workers, a psychiatric nurse, a lead psychiatrist, a pharmacist, a geriatrician, GPs, the voluntary sector, specialists for the terminally ill and their families, and home carers | Reduce demand and reliance on health and care services |  | Virgin Care |  |  |
|  |  |  |  |  | South East Coast Ambulance NHS Foundation Trust |  |  |
|  |  |  |  |  | North Hampshire Urgent Care |  |  |
|  |  |  |  |  | Hampshire county council |  |  |
|  |  |  |  |  | Surrey county council |  |  |
|  |  |  |  |  |  |  |  |
| What matters to Us   (Harrogate and Rural District CCG) | To transform the way care is provided localy with GPs, community services, hospitals, mental health and social care staff working together | Services provided by an integrated care team including GPs, community nurses, adult social care and other specialists | Reduce the need for interventions from health or social care services | Harrogate and Rural District CCG | Harrogate and District NHS Foundation Trust | 160.000 | NHS High Weald Lewes Havens CCG |
|  |  |  |  |  | Harrogate and Rural District CCG |  |  |
|  |  |  | High quality and sustainable services will offer value for money and be clinically and financially sustainable |  | North Yorkshire County council |  |  |
|  |  | A response and overnight service will provide rapid support and social care staff will work with A&E, both helping to avoid hospital admissions |  |  | Tees Esk and Wear Valley Foundation Trust |  |  |
|  | Hospital beds will be used only when they are truly needed. |  |  |  | Harrogate Borough Council |  |  |
|  |  |  | Local and personal support will help reduce uncessary hospital admissions |  | Yorkshire Health Network (representing 17 GPs) |  |  |
|  | Services supporting health and wellbeing will be better coordinated |  |  |  |  |  |  |
|  |  |  |  |  |  |  |  |
| My Life a Full Life (Isle of Wight) | To prevent ill health by promoting health and wellbeing and supporting self-care and empowered communities | Health and care services for individuals is coordinated by staff including community and care navigators and local area coordinators | Local people take better care of themselves | Isle of Wight CCG | Isle of Wight CCG | 140.000 | NHS Hastings & Rother CCG |
|  |  |  |  |  | Isle of Wight NHS Trust |  |  |
|  |  |  | People plan better for their future and take more direct control over their care |  | Isle of Wight Council |  |  |
|  | Avoid unnecessary hospital admissions and reduce inappropriate referrals to services | Introduction of new locality teams to deliver care and support in the community, with GP clinical leadership and teams of staff with a mix of skills and specialisms |  |  | One Wight Health (group of CCGs) |  |  |
|  |  |  |  |  |  |  |  |
|  |  |  | People will remain happy and healthy in their own communities, only going to hospital when they really need to |  |  |  |  |
|  |  |  |  |  |  |  |  |
|  |  |  |  |  |  |  |  |
|  |  |  |  |  |  |  |  |
|  |  |  |  |  |  |  |  |
| ^1^ NHS, 2016. New care models: Vanguards–developing a blueprint for the future of NHS and care services. London: NHS England. https://www.england.nhs.uk/wp-content/uploads/2015/11/new_care_models.pdf | | | | | | | |
| ^2^ https://www.england.nhs.uk/rightcare/products/nhs-rightcare-intelligence-tools-and-support/ . The selected comparison CCG meets two additional criteria: 1) was not a partner of any of the 50 vanguards and 2) is not a neighbour of any vanguard CCG partner. | | | | | | | |

**Table A2:** GPs closing during 2014-2018

| **Closing GP** | **GP receiving patients** | **Treatment/ Control** | **Fiscal year of closure** |
| --- | --- | --- | --- |
| N85004 | N85002 | Treatment | 2015 |
| N85011 | N85002 | Treatment | 2014 |
| N85029 | N85044 | Treatment | 2016 |
| N85041 | N85020 | Treatment | 2017 |
| N85056 | N85620 | Treatment | 2015 |
| N85058 | N85007 | Treatment | 2018 |
| N85619 | N85625 | Treatment | 2018 |
| N85635 | N85027 | Treatment | 2014 |
| Y02569 | Not clear | Treatment | 2017 |
| P87629 | P87659 | Treatment | 2015 |
| P87632 | P87020 | Treatment | 2014 |
| P87637 | P87019 | Treatment | 2014 |
| P87641 | P87026 | Treatment | 2016 |
| P87668 | Not clear | Treatment | 2018 |
| L85045 | Y01163 | Treatment | 2017 |
| L85059 | L85012 | Treatment | 2014 |
| L85602 | L85065 | Treatment | 2017 |
| L85608 | L85065 | Treatment | 2018 |
| L85616 | L85014 | Treatment | 2016 |
| Y02778 | L85064 | Treatment | 2018 |
| A84021 | A84006 | Treatment | 2018 |
| A84048 | A84009 | Treatment | 2017 |
| A84613 | A84027 & A84002 | Treatment | 2016 |
| A84618 | A84045 | Treatment | 2015 |
| C84121 | C84012 | Treatment | 2015 |
| C84675 | C84036 | Treatment | 2015 |
| C84718 | C84114 | Treatment | 2016 |
| Y02181 | Y05690 | Treatment | 2016 |
| Y02977 | C84051 | Treatment | 2015 |
| Y05159 | Not clear | Treatment | 2018 |
| C84648 | Y05369 | Treatment | 2017 |
| C84678 | C84059 | Treatment | 2015 |
| P81011 | Y01008 | Treatment | 2017 |
| P81153 | Y01008 | Treatment | 2017 |
| P81624 | Y01008 | Treatment | 2017 |
| P81056 | P81002 | Treatment | 2018 |
| P81064 | P81002 | Treatment | 2018 |
| P81091 | P81002 | Treatment | 2018 |
| P81085 | Y01008 | Treatment | 2018 |
| P81190 | P81006 | Treatment | 2018 |
| J82052 | J82049 | Treatment | 2017 |
| B82076 | B82027 | Treatment | 2016 |
| B82627 | B82013 | Treatment | 2015 |
| J84602 | Not clear | Treatment | 2017 |
| L82002 | L82041 | Control | 2016 |
| Y02596 | Not clear | Control | 2018 |
| L82019 | Y04957 | Control | 2016 |
| L82040 | Y04957 | Control | 2016 |
| L82055 | Y04957 | Control | 2015 |
| L82611 | Y04957 | Control | 2016 |
| Y04694 | Y04957 | Control | 2016 |
| L82621 | Y00969 | Control | 2016 |
| Y05021 | Y00969 | Control | 2017 |
| L82053 | Y01051 | Control | 2018 |
| B81044 | B81061 | Control | 2016 |
| B81084 | B81101 | Control | 2018 |
| B81120 | B81121 | Control | 2017 |
| B81121 | B81101 | Control | 2018 |
| B81622 | B81101 | Control | 2017 |
| B81679 | B81024 | Control | 2015 |
| M83133 | M83090 | Control | 2017 |
| M83629 | M83661 | Control | 2018 |
| M83669 | M83650 | Control | 2018 |
| M83739 | M83650 | Control | 2018 |
| M83678 | M83138 | Control | 2017 |
| M83695 | M83708 | Control | 2015 |
| M83708 | M83047 | Control | 2018 |
| Y00451 | Not clear | Control | 2016 |
| Y02868 | Not clear | Control | 2018 |
| N83016 | Not clear | Control | 2016 |
| N83636 | N83012 | Control | 2017 |
| Y02511 | Y02510 | Control | 2018 |
| B81019 | B81039 | Control | 2017 |
| B81671 | Not clear | Control | 2017 |
| B81689 | B81087 | Control | 2017 |
| B81098 | Not clear | Control | 2015 |
| B81686 | B81118 | Control | 2016 |
| F81005 | F81042 | Control | 2016 |
| F81129 | F81042 | Control | 2016 |
| F81077 | F81019 | Control | 2014 |
| F81736 | F81069 | Control | 2015 |
| Y00484 | F81095 | Control | 2018 |
| L81134 | L81130 | Control | 2015 |
| G81627 | G81053 & G81100 | Control | 2016 |
| G81692 | G81053 & G81100 | Control | 2015 |
| G81064 | G81013 | Control | 2017 |
| G81643 | G81013 | Control | 2017 |
| G81649 | G81013 | Control | 2016 |
| G81662 | G81013 | Control | 2017 |
| G81611 | G81095 | Control | 2016 |
| G81640 | G81095 | Control | 2016 |

| **Table A3.** Parallel trends tests - unmatched sample (quarters 1-9) | | |  |  |
| --- | --- | --- | --- | --- |
|  |  |  |  |  |
| **Model** | **Observations** | **ACSA rate regression** | **HHI regression** |  |
| **Aggregated PACS** | 5976 | 0.0611  [0.00,0.12]* | 0.0035  [0.002, 0.005]*** |  |
| **Wirral** | 765 | -0.0141  [-0.21,0.19] | -0.0069  [-0.010,-0.004]*** |  |
| **Mid Nottinghamshire** | 774 | 0.2858  [0.07, 0.50]*** | 0.0055  [0.002, 0.009]*** |  |
| **South Somerset** | 1188 | 0.0551  [-0.04, 0.10] | 0.0078  [0.005, 0.010]*** |  |
| **Northumberland** | 657 | 0.1567  [-0.01, 0.32]* | -0.0016  [-0.003, 0.001] |  |
| **Salford** | 801 | 0.1020  [-0.15, 0.36] | 0.0150  [0.011, 0.019]*** |  |
| **Morecambe** | 666 | -0.0876  [-.26, 0.08] | -0.0028  [-0.005, -0.001]** |  |
| **North East Hampshire** | 432 | -0.1153  [-0.28, 0.05] | -0.0047  [-0.009, -0.000]** |  |
| **Harrogate** | 324 | 0.2220  [0.06, 0.39]*** | 0.0071  [0.004, 0.010]*** |  |
| **Isle of Wight** | 369 | -0.2049  [0.43, 0.02]* | 0.0099  [0.005, 0.015]*** |  |
| 95% Confidence intervals in brackets. *p<0.10, **p<0.05, ***p<0.01. Coefficient reported is for the specific linear trend variable for treatment (treatment dummy * linear time variable). Covariates included in the models: linear time variable (one for each quarter 1-9) and a variable indicating if the quarter in question is the first, second, third or fourth in a given financial year. | | | |  |
|  |  |  |  |  |
|  |  |  |  |  |
|  |  |  |  |  |

Table A3 shows the coefficients for the specific linear trend variable for treatment (treatment dummy * linear time variable) in the ACSA rate and in the HHI (two separate regressions) for the aggregated PACS and for each of the separate PACS models uding the unmatched sample. The coefficients reported, capture the difference in the trends between intervention and comparison sites. If significant, the pre-intervention paralell trends hypothesis between intervened and comparison groups is rejected.

| **Table A4.** Matching on ACSA rate and HHI trends in quarters 1-8 | | | | | |  |
| --- | --- | --- | --- | --- | --- | --- |
| **PACS** | **Treated GPs off support** | **Treated GPs on support** | **% bias HHI trend (mean diff. as % of SD)** | **% bias ACSA rate trend (mean diff. as % of SD)** | **Comparison GPs weight Mean (SD)** |  |
| **Wirral** | 21 | 30 | -2,8 | -2,6 | 1.67 (0.8) |  |
| **Mid Nottinghamshire** | 5 | 36 | 3,5 | 3,4 | 2.00 (1.3) |  |
| **South Somerset** | 12 | 58 | 4,9 | -2.0 | 1.93 (1.3) |  |
| **Northumberland** | 6 | 35 | -4.0 | 6,5 | 2.69 (2.2) |  |
| **Salford** | 16 | 28 | -3,9 | 3,4 | 1.75 (1.3) |  |
| **Morecambe** | 9 | 27 | 6,2 | -4,4 | 1.50 (0.6) |  |
| **North East Hampshire** | 14 | 9 | -1,7 | 5,2 | 1.29 (0.5) |  |
| **Harrogate^ᵠ^** | 1 | 16 | -0,5 | NA | 2.29 (1.5) |  |
| **Isle of Wight** | 5 | 11 | 6,8 | 14,2 | 1.38 (0.7) |  |
| Results from Mahalanobis matching on the HHI and ACSA rate trends with a caliper of 0.25. ᵠIn Harrogate, matching on the HHI trend only was preferred. % bias is the mean difference expressed as % of the standard deviation. GPs weights is the mean frequency with wich each comparison GP in the common support was used as match for a treated GP. | | | | | |  |
|  |  |  |  |  |  |  |
|  |  |  |  |  |  |  |

| **Table A5.** Parallel trends tests - unmatched sample (quarters 1-8) | | | |  |
| --- | --- | --- | --- | --- |
|  |  |  |  |  |
| **Model** | **Observations** | **ACSA rate regression** | **HHI regression** |  |
| **Aggregated PACS** | 4000 | 0.0128  [-0.06,0.09] | 0.0001  [-0.001, 0.001] |  |
| **Wirral** | 480 | -0.0133  [-0.24,0.21] | -0.0003  [-0.004,-0.004] |  |
| **Mid Nottinghamshire** | 576 | 0.0192  [-0.19, 0.23] | -0.0004  [-0.003,-0.004] |  |
| **South Somerset** | 928 | -0.0073  [-0.11, 0.10] | 0.0005  [-0.002, 0.003] |  |
| **Northumberland** | 560 | 0.0284  [-0.15, 0.21] | -0.0002  [-0.002, 0.001] |  |
| **Salford** | 448 | 0.0279  [-0.31, 0.37] | 0.0004  [-0.004, 0.004] |  |
| **Morecambe** | 432 | -0.0170  [-0.22, 0.19] | 0.0004  [-0.003, 0.003] |  |
| **North East Hampshire** | 144 | 0.0200  [-0.32, 0.36] | 0.0003  [-0.006, 0.006] |  |
| **Harrogate** | 256 | 0.0856  [-0.12, 0.29] | 0.0000  [-0.003, 0.003] |  |
| **Isle of Wight** | 176 | 0.0437  [-0.23, 0.31] | 0.0006  [-0.006, 0.007] |  |
| 95% Confidence intervals in brackets. *p<0.10, **p<0.05, ***p<0.01. Coefficient reported is for the specific linear trend variable for treatment (treatment dummy * linear time variable). Covariates included in the models: linear time variable (one for each quarter 1-9) and a variable indicating if the quarter in question is the first, second, third or fourth in a given financial year. | | | |  |
|  |  |  |  |  |
|  |  |  |  |  |
|  |  |  |  |  |

| Table A5 shows the coefficients for the specific linear trend variable for treatment (treatment dummy * linear time variable) in the ACSA rate and in the HHI (two separate regressions) for the aggregated PACS and for each of the separate PACS models using the matched sample. The coefficients reported, capture the difference in the trends between intervention and comparison sites. If significant, the pre-intervention paralell trends hypothesis between intervened and comparison groups is rejected. |  |
| --- | --- |
|  |  |
|  |  |
|  |  |

| **Table A6_a.** Mediation analysis excluding anticipation period: mediated effect of PACS on ACSAs through integrated care - unmatched sample | | | | | | | |  |
| --- | --- | --- | --- | --- | --- | --- | --- | --- |
| **Model** | **Observations** | **PACS effect on HHI  (βa )** | **Relation between HHI and ACSA  (βb)** | **Indirect effect  (βa*βb)** | **Direct effect  (β'c)** | **Treatment effect  (βc)** | **Total effect  [(βa*βb) + β'c)]** |  |
| **Aggregated PACSᵠ** | 11952 | 0.05  [0.00,0.09]** | 2.20  [0.34, 4.06]** | 0.10  [-0.05, 0.26] | -0.15  [-0.89, 0.59] | -0.05  [-0.78, 0.69] | -0.05  [-0.78, 0.69] |  |
| **Wirral** | 1530 | 0.02  [0.01, 0.04]*** | 0.80  [-3.18, 4.78] | 0.02  [-0.08, 0.12] | -0.04  [-0.83, 0.76] | -0.02  [-0.79, 0.76] | -0.02  [-0.79, 0.76] |  |
| **Mid Nottinghamshire** | 1548 | 0.14  [0.12, 0.16]*** | 3.23  [0.16, 6.30]** | 0.44  [0.02, 0.86]** | 1.05  [0.18, 1.92]** | 1.49  [0.68, 2.31]*** | 1.49  [0.68, 2.31]*** |  |
| **South Somerset** | 2376 | 0.08  [0.07, 0.09]*** | -0.06  [-1.92, 1.81] | -0.00  [-0.15, 0.14] | -0.04  [-0.42, 0.34] | -0.04  [-0.40, 0.31] | -0.04  [-0.40, 0.31] |  |
| **Northumberland** | 1314 | -0.02  [-0.03, -0.01]*** | 0.66  [-3.47, 4.79] | -0.01  [-0.11, 0.08] | 0.16  [-0.44, 0.75] | 0.14  [-0.46, 0.74] | 0.14  [-0.46, 0.74] |  |
| **Salford** | 1602 | 0.12  [0.11, 0.13]*** | 3.18  [-1.83, 8.19] | 0.38  [-0.23, 0.99] | -1.78  [-2.95, -0.62]*** | -1.40  [-2.39, -0.41]*** | -1.40  [-2.39, -0.41]*** |  |
| **Morecambe** | 1332 | 0.00  [-0.01, 0.01] | 4.72  [-0.75, 10.20] | 0.00  [-0.05, 0.05] | -1.97  [-2.58, -1.36] | -1.97  [-2.57, -1.36]*** | -1.97  [-2.57, -1.36]*** |  |
| **North East Hampshire** | 864 | 0.06  [0.05, 0.08]*** | 2.46  [-0.69, 5.62] | 0.15  [-0.04, 0.34] | 0.38  [-0.29, 1.05] | 0.53  [-0.12, 1.17] | 0.53  [-0.12, 1.17] |  |
| **Harrogate** | 648 | 0.02  [0.01, 0.03]*** | 2.18  [-2.13, 6.48] | 0.04  [-0.05, 0.14] | 0.06  [-0.55, 0.67] | 0.10  [-0.50, 0.71] | 0.10  [-0.50, 0.71] |  |
| **Isle of Wight** | 738 | -0.17  [-0.19,-0.15]*** | -3.13  [-6.61,0.36]* | 0.53  [-0.07, 1.13]* | 0.48  [-0.36,1.31] | 1.01  [0.33, 1.68]*** | 1.01  [0.33, 1.68]*** |  |
| 95% confidence intervals in brackets (built with bootstapped standard errors, 1000 replications). *p<0.10, **p<0.05, ***p<0.01. ᵠConfidence intervals built with clustered standard errors at the PACS level and bootstrapped with 1000 replications. | | | | | | | |  |
|  |  |  |  |  |  |  |  |  |

| **Table A6_b.** Mediation analysis excluding anticipation period with one quarter lag between HHI and ACSA rate - unmatched sample | | | | | | | |  |
| --- | --- | --- | --- | --- | --- | --- | --- | --- |
| **Model** | **Observations** | **PACS effect on HHI  (βa )** | **Relation between HHI and ACSA  (βb)** | **Indirect effect  (βa*βb)** | **Direct effect  (β'c)** | **Treatment effect  (βc)** | **Total effect  [(βa*βb) + β'c)]** |  |
| **Aggregated PACSᵠ** | 10624 | 0.05  [0.00, 0.09]** | 1.43  [-1.41, 4.27] | 0.07  [-0.11, 0.25] | -0.12  [-0.81, 0.56] | -0.05  [-0.80, 0.69] | -0.05  [-0.80, 0.69] |  |
| **Wirral** | 1360 | 0.02  [0.01, 0.04]*** | -3.88  [-8.93, 1.18] | -0.08  [-0.21, 0.04] | 0.02  [-0.78, 0.82] | -0.07  [-0.86, 0.72] | -0.07  [-0.86, 0.72] |  |
| **Mid Nottinghamshire** | 1376 | 0.13  [0.11, 0.15]*** | 5.66  [2.45, 8.87]*** | 0.75  [0.32, 1.18]*** | 0.71  [-0.18, 1.60] | 1.46  [0.53, 2.38]*** | 1.46  [0.53, 2.38]*** |  |
| **South Somerset** | 2112 | 0.08  [0.07, 0.09]*** | 0.67  [-1.58, 2.93] | 0.05  [-0.13, 0.24] | -0.11  [-0.55, 0.33] | -0.06  [-0.44, 0.33] | -0.06  [-0.44, 0.33] |  |
| **Northumberland** | 1168 | -0.02  [-0.03,  -0.01]*** | -2.10  [-7.21, 3.01] | 0.05  [-0.08, 0.17] | 0.16  [-0.49, 0.81] | 0.21  [-0.46, 0.88] | 0.21  [-0.46, 0.88] |  |
| **Salford** | 1424 | 0.12  [0.11, 0.13]*** | -1.31  [-6.54, 3.91] | -0.16  [-0.78, 0.47] | -1.11  [-2.42, 0.19]* | -1.27  [-2.34, -0.19]** | -1.27  [-2.34, -0.19]** |  |
| **Morecambe** | 1184 | -0.00  [-0.01, 0.01] | -3.79  [-10.25, 2.67] | 0.00  [-0.04, 0.06] | -2.16  [-2.82, -1.50]*** | -2.15  [-2.81, -1.49]*** | -2.15  [-2.81, -1.49]*** |  |
| **North East Hampshire** | 768 | 0.05  [0.04, 0.07]*** | 0.43  [-2.83, 3.69] | 0.02  [-0.15, 0.20] | 0.43  [-0.29, 1.15] | 0.45  [-0.25, 1.15] | 0.45  [-0.25, 1.15] |  |
| **Harrogate** | 576 | 0.03  [0.01, 0.04]*** | 8.66  [4.35, 12.98]*** | 0.22  [0.07, 0.37]*** | -0.14  [-0.79, 0.50] | 0.08  [-0.57, 0.72] | 0.08  [-0.57, 0.72] |  |
| **Isle of Wight** | 656 | -0.16  [-0.18, -0.14]*** | 1.00  [-2.82, 4.83] | -0.16  [-0.76, 0.44] | 1.28  [0.34, 2.21]*** | 1.12  [0.41, 1.83]*** | 1.12  [0.41, 1.83]*** |  |
| 95% confidence intervals in brackets (built with bootstapped standard errors, 1000 replications). *p<0.10, **p<0.05, ***p<0.01. ᵠConfidence intervals built with clustered standard errors at the PACS level and bootstrapped with 1000 replications. | | | | | | | |  |
|  |  |  |  |  |  |  |  |  |

| **Table A7.** Mediation analysis excluding anticipation period: mediated effect of PACS on ACSAs through integrated care - including covariates | | | | | | | |  |
| --- | --- | --- | --- | --- | --- | --- | --- | --- |
| **Model** | **Observations** | **PACS effect on HHI  (βa )** | **Relation between HHI and ACSA  (βb)** | **Indirect effect  (βa*βb)** | **Direct effect  (β'c)** | **Treatment effect  (βc)** | **Total effect  [(βa*βb) + β'c)]** |  |
| **Aggregated PACSᵠ** | 9000 | 0.04  [0.01, 0.08]** | 1.85  [-0.43, 4.13] | 0.08  [-0.06, 0.21] | -0.30  [-1.15, 0.54] | 0.23  [-1.06, 0.06] | 0.23  [-1.06, 0.06] |  |
| **Wirral** | 1080 | 0.03  [0.01, 0.05]*** | 0.87  [-3.33, 5.07] | 0.03  [-0.11, 0.17] | 0.58  [-0.41, 1.57] | 0.60  [-0.39, 1.60] | 0.60  [-0.39, 1.60] |  |
| **Mid Nottinghamshire** | 1296 | 0.09  [0.07, 0.10]*** | 2.82  [0.02, 5.63]** | 0.24  [-0.00, 0.49]** | 1.69  [0.95, 2.42]*** | 1.93  [1.24, 2.62]*** | 1.93  [1.24, 2.62]*** |  |
| **South Somerset** | 2088 | 0.08  [0.07, 0.09]*** | 0.19  [-2.08, 2.46] | 0.02  [-0.17, 0.20] | -0.72  [-1.21, -0.23]*** | -0.70  [-1.15, -0.26]*** | -0.70  [-1.15, -0.26]*** |  |
| **Northumberland** | 1260 | -0.03  [-0.03, -0.02]*** | -0.71  [-4.56, 3.13] | 0.02  [-0.09, 0.13] | 0.29  [-0.31, 0.90] | 0.31  [-0.29, 0.91] | 0.31  [-0.29, 0.91] |  |
| **Salford** | 1008 | 0.11  [0.10, 0.13]*** | 5.03  [-1.40, 11.47] | 0.58  [-0.16, 1.31] | -1.82  [-3.28, -0.37]** | -1.25  [-2.46, -0.04]** | -1.25  [-2.46, -0.04]** |  |
| **Morecambe** | 972 | -0.01  [-0.02, -0.00]** | 6.55  [2.35, 10.74]** | -0.09  [-0.19, 0.01]* | -1.91  [-2.54, -1.29]*** | -2.00  [-2.63, -1.38]*** | -2.00  [-2.63, -1.38]*** |  |
| **North East Hampshire** | 324 | 0.10  [0.07, 0.13]*** | -0.97  [-9.41, 7.47] | -0.10  [-0.95, 0.75] | 2.05  [0.24, 3.87]** | 1.96  [0.46, 3.46]*** | 1.96  [0.46, 3.46]*** |  |
| **Harrogate** | 576 | 0.00  [-0.01, 0.02] | -1.72  [-6.84, 3.40] | -0.00  [-0.06, 0.04] | -0.07  [-1.04, 0.90] | -0.08  [-1.05, 0.89] | -0.08  [-1.05, 0.89] |  |
| **Isle of Wight** | 396 | -0.14  [-0.17, -0.11]*** | -2.97  [-7.75, 1.81] | 0.40  [-0.26, 1.07] | 0.04  [-1.23, 1.15] | -0.04  [-0.63, 1.37] | -0.04  [-0.63, 1.37] |  |
| 95% confidence intervals in brackets (built with bootstapped standard errors, 1000 replications). *p<0.10, **p<0.05, ***p<0.01. ᵠConfidence intervals built with clustered standard errors at the PACS level and bootstrapped with 1000 replications. Covariates included in the models: GP list size, the proportion of patients registered with the practice aged 65 and over, the proportion of patients that were male, the number of FTE GPs in the practice and prevalence rates of hypertension, diabetes, heart failure and obesity amongst the registered population. | | | | | | | |  |
|  |  |  |  |  |  |  |  |  |
|  |  |  |  |  |  |  |  |  |

| **Table A8_a.** Mediation analysis excluding anticipation period with one semester lag between HHI and ACSA rate | | | | | | | |  |
| --- | --- | --- | --- | --- | --- | --- | --- | --- |
| **Model** | **Observations** | **PACS effect on HHI  (βa )** | **Relation between HHI and ACSA  (βb)** | **Indirect effect  (βa*βb)** | **Direct effect  (β'c)** | **Treatment effect  (βc)** | **Total effect  [(βa*βb) + β'c)]** |  |
| **Aggregated PACSᵠ** | 7000 | 0.04  [0.01, 0.08]** | 0.76  [-2.22, 3.74] | 0.03  [-0.11, 0.17] | -0.35  [-1.10, 0.40] | -0.31  [-1.10, 0.47] | -0.31  [-1.10, 0.47] |  |
| **Wirral** | 840 | 0.04  [0.03, 0.06]*** | -4.21  [-8.96, 0.54]* | -0.18  [-0.40, 0.04] | 0.25  [-0.66, 1.17] | 0.07  [-0.81, 0.94] | 0.07  [-0.81, 0.94] |  |
| **Mid Nottinghamshire** | 1008 | 0.09  [0.07, 0.11]*** | 4.78  [1.99, 7.57]*** | 0.43  [0.17, 0.69]*** | 0.93  [0.10, 1.76]** | 1.36  [0.56, 2.16]*** | 1.36  [0.56, 2.16]*** |  |
| **South Somerset** | 1624 | 0.09  [0.08, 0.10]*** | 0.49  [-2.05, 3.04] | 0.04  [-0.18, 0.27] | -0.40  [-0.85, 0.05]* | -0.36  [-0.75, 0.04]* | -0.36  [-0.75, 0.04]* |  |
| **Northumberland** | 980 | -0.02  [-0.03, -0.1]*** | -6.28  [-10.23, -2.34]*** | 0.12  [0.03, 0.21]*** | -0.01  [-0.64, 0.63] | 0.11  [-0.52, 0.74] | 0.11  [-0.52, 0.74] |  |
| **Salford** | 784 | 0.11  [0.09, 0.12]*** | 4.33  [-3.36, 12.02] | 0.46  [-0.35, 1.27] | -2.45  [-4.08, -0.82]*** | -1.99  [-3.26, -0.72]*** | -1.99  [-3.26, -0.72]*** |  |
| **Morecambe** | 756 | -0.01  [-0.02, 0.00] | -5.75  [-10.43, -1.06]** | 0.05  [-0.03, 0.12] | -2.29  [-2.97, -1.61] | -2.24  [-2.93, -1.55]*** | -2.24  [-2.93, -1.55]*** |  |
| **North East Hampshire** | 252 | 0.09  [0.07, 0.11]*** | 2.63  [-5.62, 10.88] | -0.24  [-0.51, 0.99] | 0.79  [-2.05, 0.46] | -0.55  [-1.63, 0.52] | -0.55  [-1.63, 0.52] |  |
| **Harrogate** | 448 | -0.01  [-0.02, 0.01] | 4.43  [-0.48, 9.34]* | -0.03  [-0.10, 0.04] | -0.11  [-0.84, 0.63] | -0.13  [-0.86, 0.60] | -0.13  [-0.86, 0.60] |  |
| **Isle of Wight** | 308 | -0.13  [-0.16, -0.10]*** | 2.84  [-1.91, 7.59] | -0.38  [-1.01, 0.26] | 1.34  [0.27, 2.41]** | 0.96  [0.04, 1.89]** | 0.96  [0.04, 1.89]** |  |
| 95% confidence intervals in brackets (built with bootstapped standard errors, 1000 replications). *p<0.10, **p<0.05, ***p<0.01. ᵠConfidence intervals built with clustered standard errors at the PACS level and bootstrapped with 1000 replications. | | | | | | | |  |
|  |  |  |  |  |  |  |  |  |

| **Table A8_b.** Mediation analysis excluding anticipation period with one year lag between HHI and ACSA rate | | | | | | |  |  |
| --- | --- | --- | --- | --- | --- | --- | --- | --- |
| **Model** | **Observations** | **PACS effect on HHI  (βa )** | **Relation between HHI and ACSA  (βb)** | **Indirect effect  (βa*βb)** | **Direct effect  (β'c)** | **Treatment effect  (βc)** | **Total effect  [(βa*βb) + β'c)]** |  |
| **Aggregated PACSᵠ** | 5000 | 0.05  [0.01, 0.08]** | -0.94  [-3.08, 1.19] | -0.04  [-0.12, 0.03] | -0.22  [-0.98, 0.54] | -0.26  [-1.03, 0.50] | -0.26  [-1.03, 0.50] |  |
| **Wirral** | 600 | 0.05  [0.03, 0.06]*** | -1.36  [-7.12, 4.40] | -0.06  [-0.32, 0.20] | 0.05  [-1.04, 1.15] | -0.01  [-1.04, 1.03] | -0.01  [-1.04, 1.03] |  |
| **Mid Nottinghamshire** | 720 | 0.09  [0.06, 0.11]*** | 0.14  [-3.20, 3.48] | 0.01  [-0.27, 0.29] | 1.38  [0.49, 2.27]*** | 1.39  [0.55, 2.24]*** | 1.39  [0.55, 2.24]*** |  |
| **South Somerset** | 1160 | 0.09  [0.08, 0.10]*** | 1.15  [-2.09, 4.39] | 0.11  [-0.19, 0.40] | -0.23  [-0.82, 0.36] | -0.12  [-0.61, 0.36] | -0.12  [-0.61, 0.36] |  |
| **Northumberland** | 700 | -0.02  [-0.03, -0.01]*** | -3.33  [-8.03, 1.37] | 0.07  [-0.04, 0.18] | 0.06  [-0.67, 0.78] | 0.13  [-0.58, 0.84] | 0.13  [-0.58, 0.84] |  |
| **Salford** | 560 | 0.10  [0.08, 0.12]*** | -2.19  [-11.35, 6.97] | -0.23  [-1.17, 0.72] | -1.64  [-3.53, 0.26]* | -1.86  [-3.39, -0.33]** | -1.86  [-3.39, -0.33]** |  |
| **Morecambe** | 540 | 0.00  [-0.01, 0.01] | -5.99  [-11.89, -0.09]** | 0.00  [-0.10, 0.10] | -2.15  [-2.98, -1.32]*** | -2.15  [-2.98, -1.31]*** | -2.15  [-2.98, -1.31]*** |  |
| **North East Hampshire** | 180 | 0.08  [0.05,0.11]*** | 2.43  [-8.17, 13.04] | 0.20  [-0.68, 1.08] | -0.96  [-2.53, 0.62] | -0.76  [-2.08, 0.56] | -0.76  [-2.08, 0.56] |  |
| **Harrogate** | 320 | 0.00  [-0.02, 0.01] | 3.18  [-2.79, 9.16] | -0.01  [-0.08, 0.07] | -0.11  [-0.97, 0.75] | -0.12  [-0.97, 0.74] | -0.12  [-0.97, 0.74] |  |
| **Isle of Wight** | 220 | -0.09  [-0.13, -0.06]*** | -4.14  [-9.17, 0.89] | 0.38  [-0.10, 0.86] | 0.13  [-1.10, 1.36] | 0.51  [-0.64, 1.67] | 0.51  [-0.64, 1.67] |  |
| 95% confidence intervals in brackets (built with bootstapped standard errors, 1000 replications). *p<0.10, **p<0.05, ***p<0.01. ᵠConfidence intervals built with clustered standard errors at the PACS level and bootstrapped with 1000 replications. | | | | | | | |  |
|  |  |  |  |  |  |  |  |  |

| **Table A9_a.** Test of mediator-treatment interaction effect on ACSA rate - no lag | | |  |  |  |
| --- | --- | --- | --- | --- | --- |
| **Model** | **Observations** | **βb** | **β'c** | **Interaction  HHI * D_it** |  |
| **Aggregated PACSᵠ** | 9000 | 1.95  [-0.40, 4.29] | 0.37  [-1.33, 2.08] | -1.04  [-3.71, 1.64] |  |
| **Wirral** | 1080 | -0.75  [-5.17, 3.67] | -1.67  [-6.19, 2.85] | 4.14  [-5.74, 14.01] |  |
| **Mid Nottinghamshire** | 1296 | 1.10 [-1.46, 3.67] | 2.79  [-0.17, 5.75] | 0.53  [-3.58, 4.64] |  |
| **South Somerset** | 2088 | -0.19  [-2.52, 2.14] | -0.95  [-1.78, -0.12]** | 0.99  [-0.35, 2.33] |  |
| **Northumberland** | 1260 | 0.58  [-4.27, 5.43] | 0.01  [-2.31, 2.32] | -1.44  [-4.89, 2.00] |  |
| **Salford** | 1008 | 7.23  [-0.06, 14.53]** | -0.38  [-5.13, 4.37] | -4.98  [-15.99, 6.03] |  |
| **Morecambe** | 972 | 5.65  [1.55, 9.75]*** | -1.49  [-4.37, 1.40] | -0.51  [-4.12, 3.10] |  |
| **North East Hampshire** | 324 | 4.27  [-4.41, 12.96] | 10.22  [-0.92, 21.36]* | -14.15  [-29.58, 1.28]* |  |
| **Harrogate** | 576 | 0.17  [-4.97, 5.32] | -2.41  [-5.98, 1.17] | 3.14  [-1.64, 7.91] |  |
| **Isle of Wight** | 396 | -5.82  [-12.89, 1.24] | -4.14  [-11.84, 3.56] | 5.99  [-4.63, 16.62] |  |
| 95% confidence intervals in brackets (built with bootstapped standard errors, 1000 replications). *p<0.10, **p<0.05, ***p<0.01. ᵠConfidence intervals built with clustered standard errors at the PACS level and bootstrapped with 1000 replications. | | | | |  |
|  |  |  |  |  |  |
|  |  |  |  |  |  |

| **Table A9_b.** Test of mediator-treatment interaction effect on ACSA rate - one quarter lag between HHI and ACSA rate | | | | |  |
| --- | --- | --- | --- | --- | --- |
| **Model** | **Observations** | **βb** | **β'c** | **Interaction  HHI * D_it** |  |
| **Aggregated PACSᵠ** | 8000 | 1.42  [-1.47, 4.30] | 0.38  [-1.09, 1.85] | -1.09  [-3.77, 1.59] |  |
| **Wirral** | 960 | -5.66  [-10.18, -1.13] | -5.29  [-10.11, -0.47] | 11.84  [1.27, 22.40]** |  |
| **Mid Nottinghamshire** | 1152 | 4.74  [1.83, 7.65]*** | 0.16  [-2.15, 2.47] | 1.74  [-1.93, 5.40] |  |
| **South Somerset** | 1856 | 1.04  [-1.64, 3.72] | -0.59  [-1.54, 0.35] | 0.18  [-1.33, 1.69] |  |
| **Northumberland** | 1120 | -0.60  [-4.29, 3.09] | -1.05  [-3.47, 1.36] | 3.30  [-1.73, 8.33] |  |
| **Salford** | 896 | 1.54  [-3.11, 6.19] | 2.37  [-4.66, 9.40] | -7.90  [-18.55, 2.76] |  |
| **Morecambe** | 864 | -0.85  [-5.12, 3.42] | -1.07  [-3.98, 1.85] | -1.48  [-5.02, 2.06] |  |
| **North East Hampshire** | 288 | -5.23  [-14.02, 3.57] | 1.17  [-10.46, 12.81] | -1.08  [-17.18, 15.02] |  |
| **Harrogate** | 512 | 13.86  [9.44, 18.28]*** | -1.81  [-5.04, 1.42] | 2.51  [-1.83, 6.84] |  |
| **Isle of Wight** | 352 | -5.81  [-14.09, 2.47] | -5.05  [-12.35, 2.25] | 8.76  [-2.46, 19.99] |  |
| 95% confidence intervals in brackets (built with bootstapped standard errors, 1000 replications). *p<0.10, **p<0.05, ***p<0.01. ᵠConfidence intervals built with clustered standard errors at the PACS level and bootstrapped with 1000 replications. | | | | |  |
|  |  |  |  |  |  |
|  |  |  |  |  |  |

| **Table A10.** Mediation analysis excluding anticipation period: mediated effect of PACS on ACSAs through integrated care - excluding North East Hampshire and Wirral PACS | | | | | | | |  |
| --- | --- | --- | --- | --- | --- | --- | --- | --- |
| **Model** | **Observations** | **PACS effect on HHI  (βa )** | **Relation between HHI and ACSA  (βb)** | **Indirect effect  (βa*βb)** | **Direct effect  (β'c)** | **Treatment effect  (βc)** | **Total effect  [(βa*βb) + β'c)]** |  |
| **Aggregated PACS^ᵠ^ (simultaneous model)** | 8676 | 0.04  [0.01, 0.08]** | 1.96  [-0.29, 4.20]* | 0.08  [-0.06, 0.22] | -0.25  [-1.09, 0.60] | -0.16  [-1.00, 0.67] | -0.16  [-1.00, 0.67] |  |
| **Aggregated PACS^ᵠᵠ^  (one-quarter lag model)** | 7040 | 0.05  [0.00, 0.09]* | 1.68  [-1.41, 4.77] | 0.08  [-0.09, 0.24] | -0.31  [-1.23, 0.62] | -0.23  [-1.21, 0.74] | -0.23  [-1.21, 0.74] |  |
| 95% confidence intervals in brackets (built with clustered standard errors at PACS level and bootstrapped with 1000 replications). *p<0.10, **p<0.05, ***p<0.01.  ᵠ Excludes North East Hampshire PACS. ᵠᵠ Excludes Wirral PACS | | | | | | | |  |
|  |  |  |  |  |  |  |  |  |

**Figure A1.** ACSA rate and HHI trends - unmatched sample


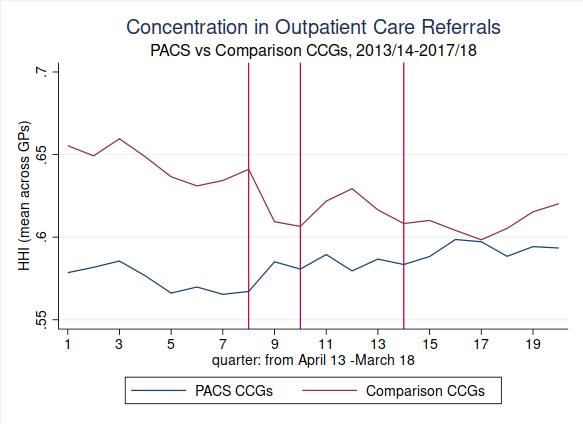

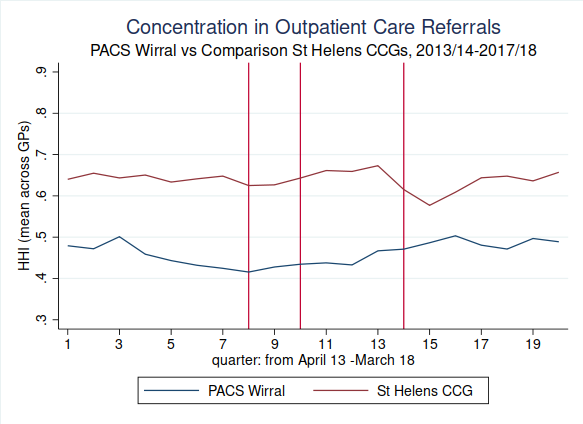

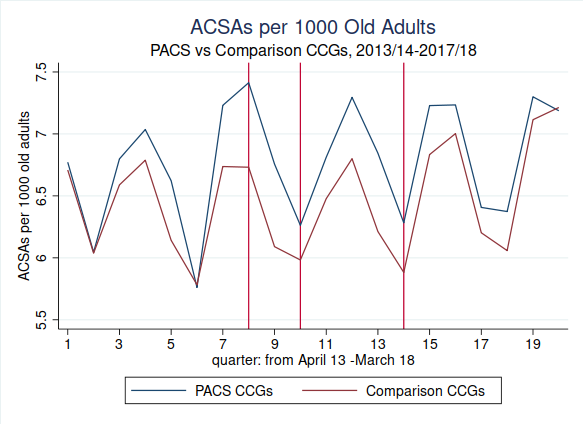

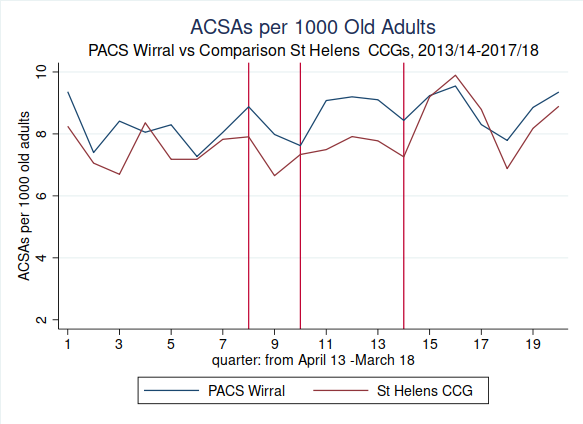


**Figure A1.** ACSA rate and HHI trends - unmatched sample – continued


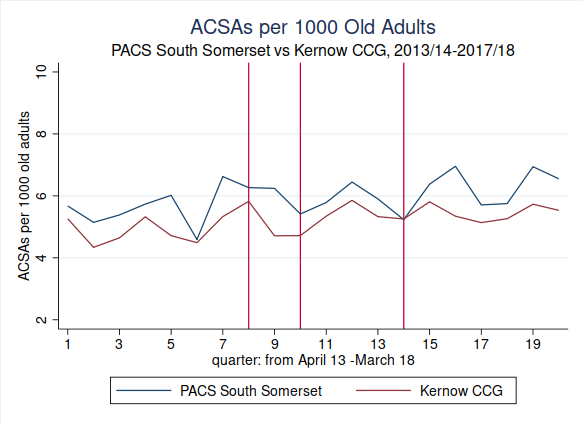

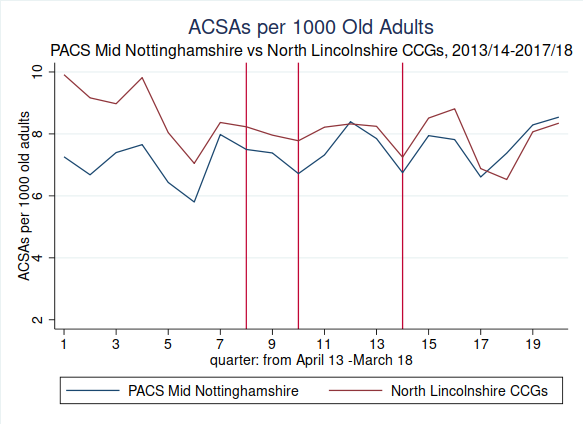


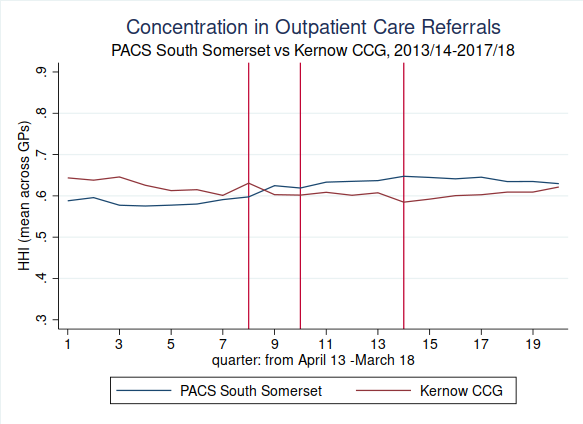

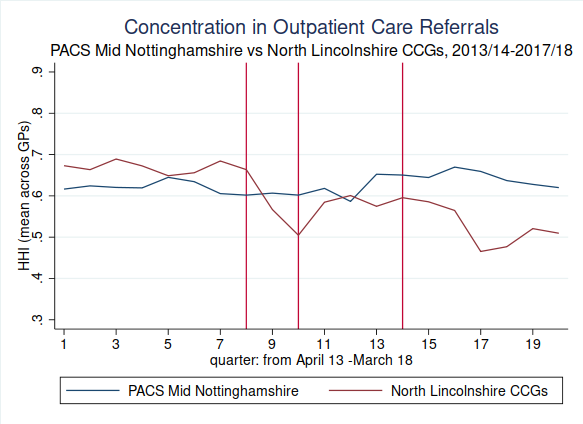


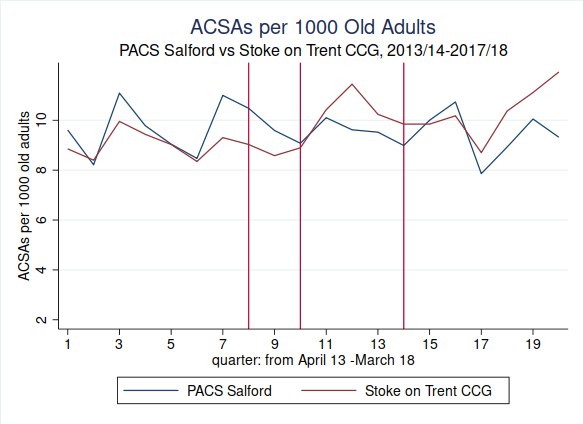
**Figure A1.** ACSA rate and HHI trends - unmatched sample – continued


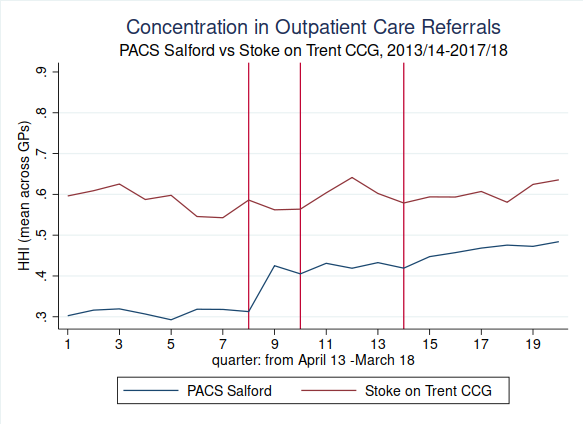

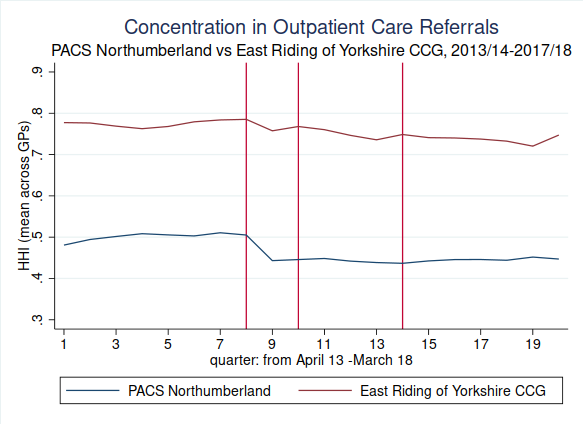

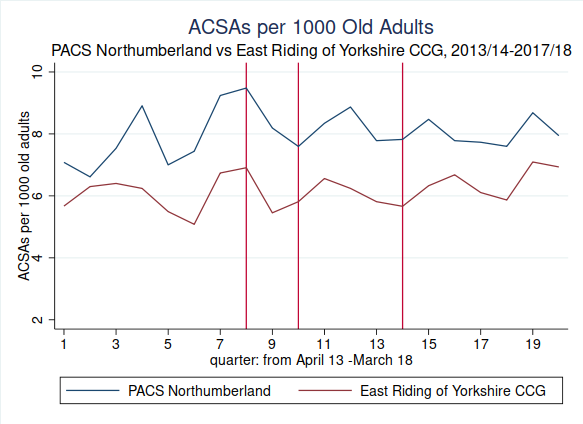


**Figure A1.** ACSA rate and HHI trends - unmatched sample – continued


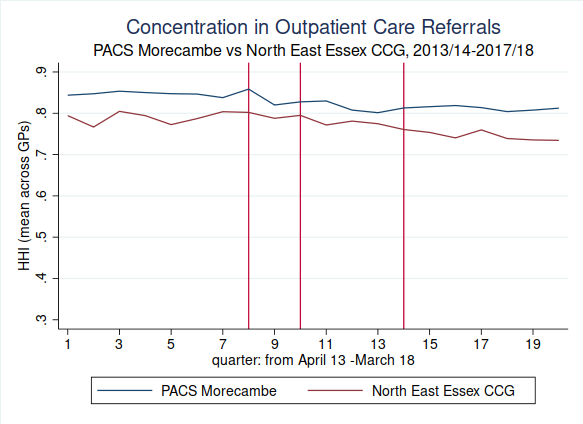

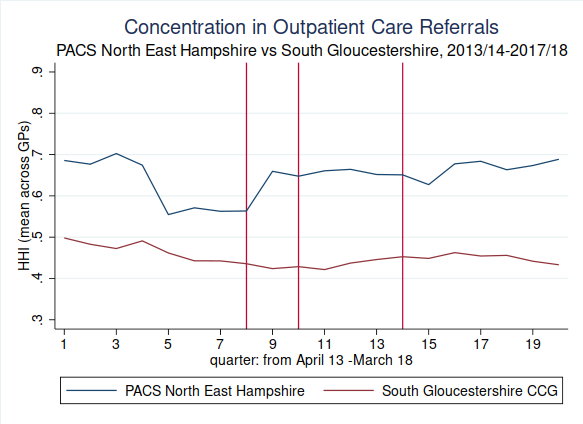

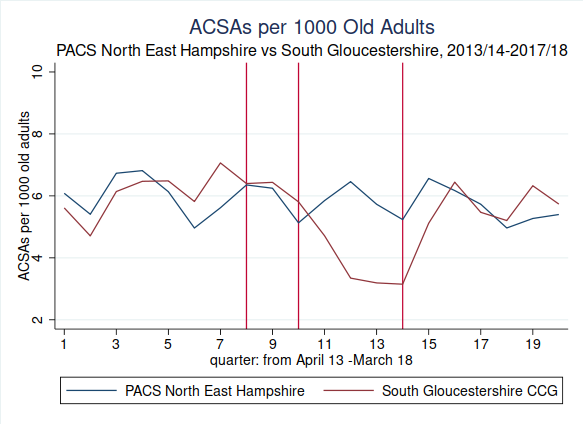

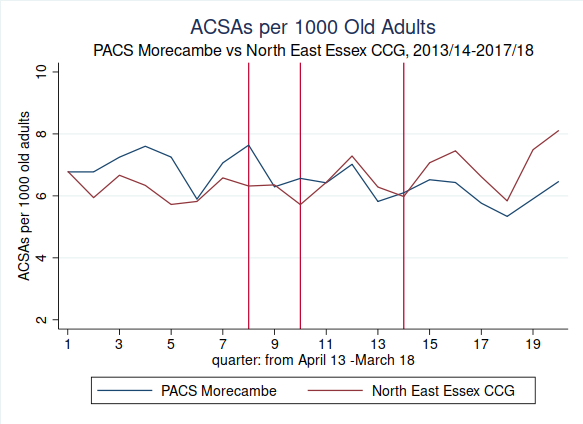


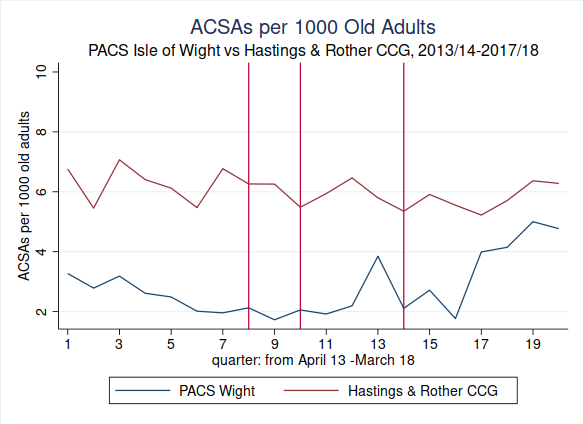
**Figure A1.** ACSA rate and HHI trends - unmatched sample – continued


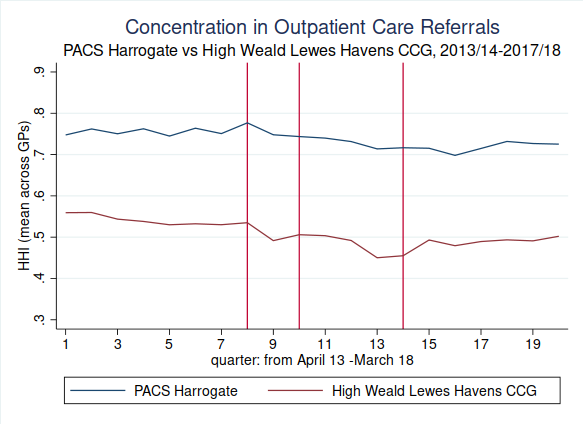

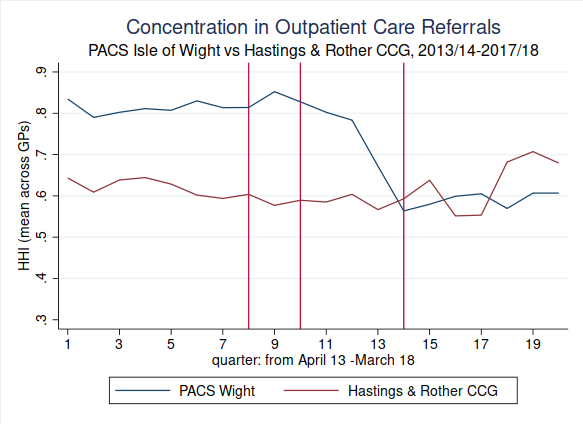

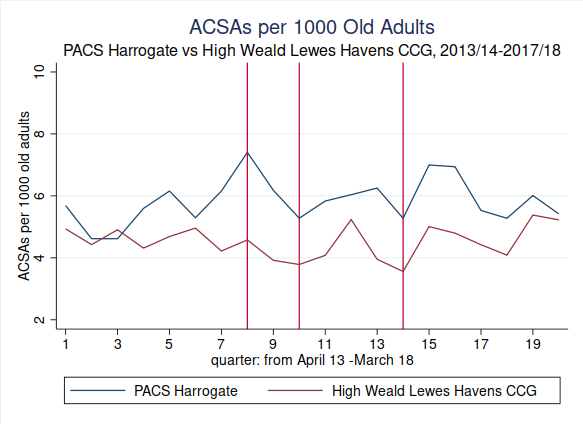


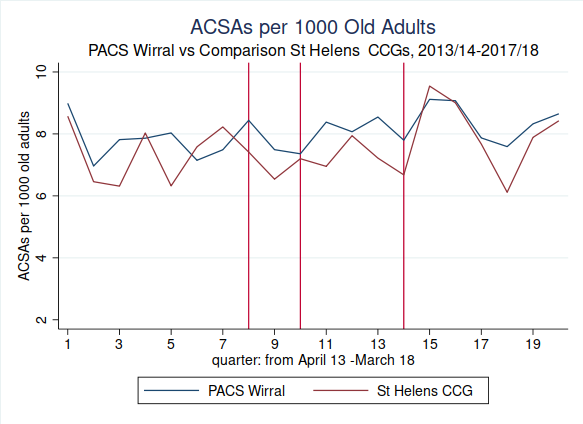
**Figure A2. ACSA rate and HHI trends - matched sample**


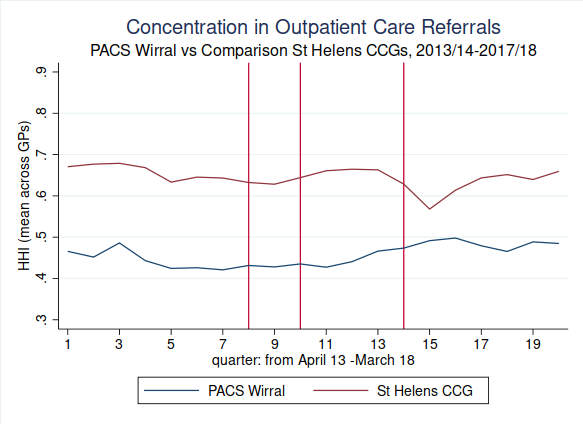

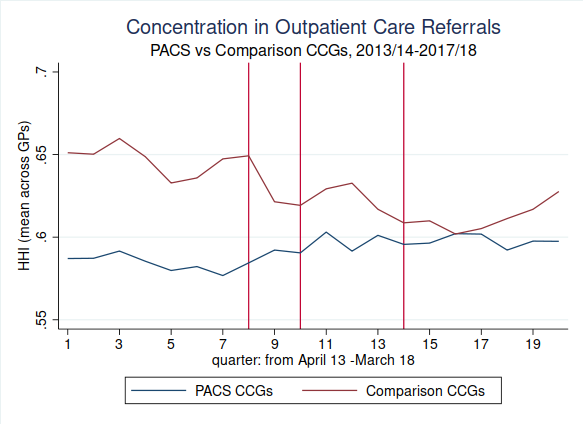

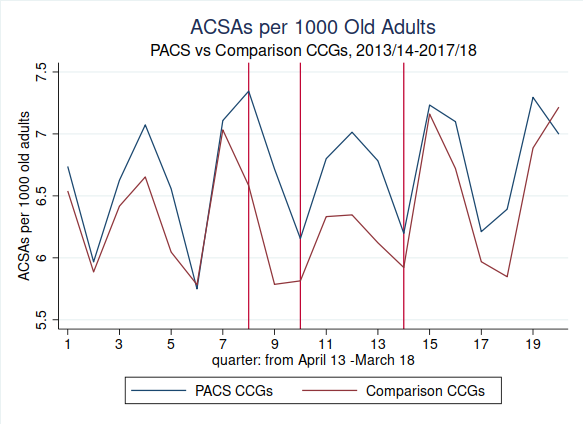


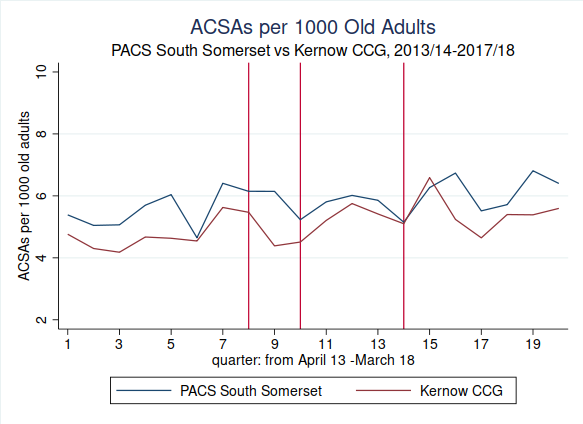
**Figure A2. ACSA rate and HHI trends - matched sample - continued**


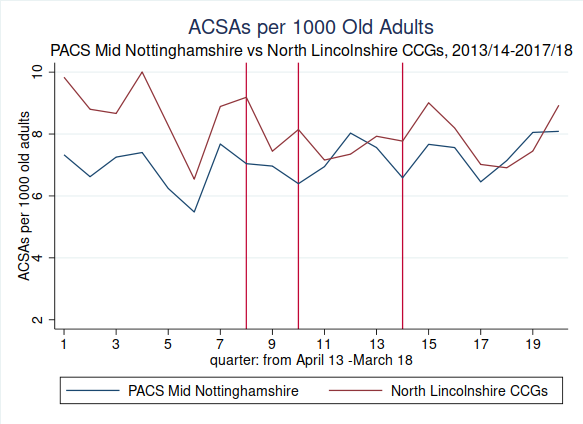


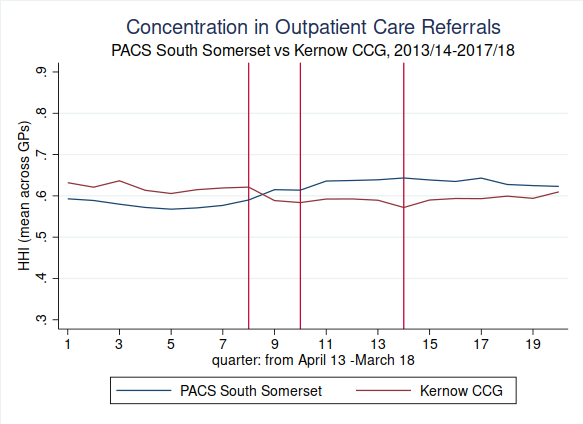

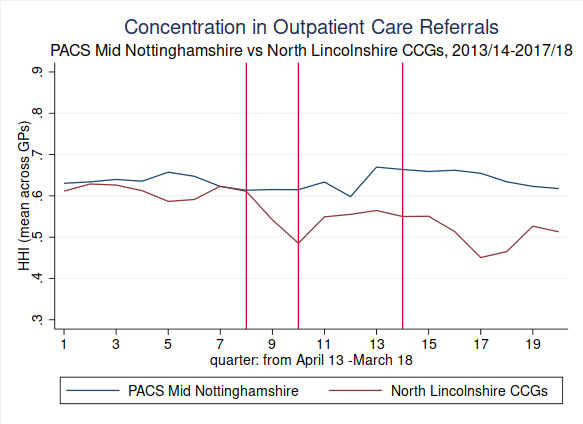


**Figure A2. ACSA rate and HHI trends - matched sample – continued**


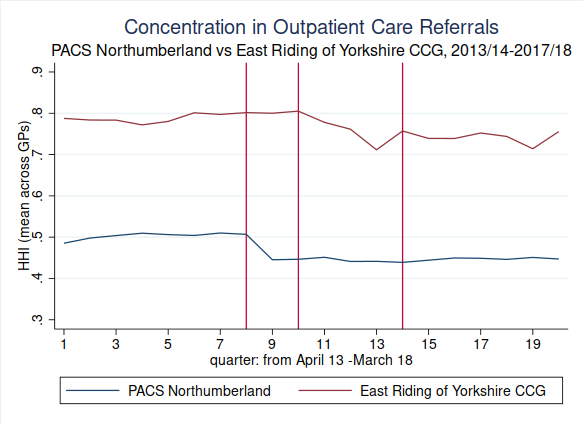

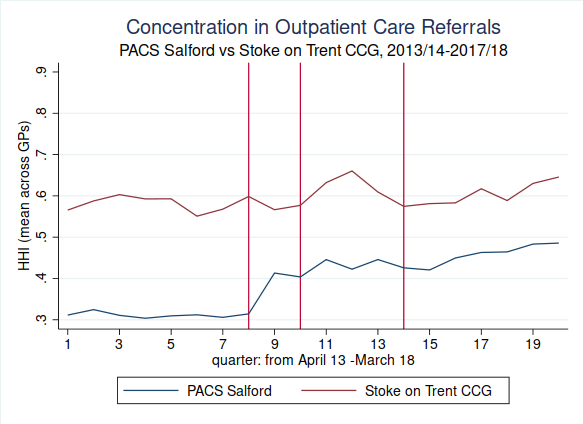

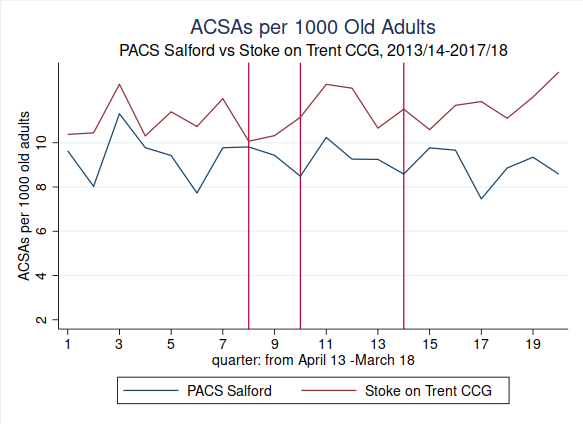

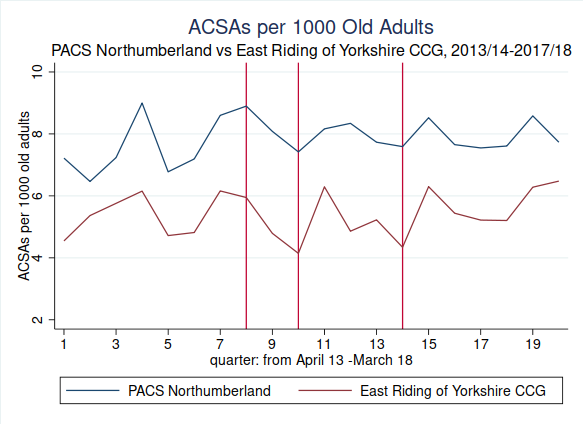


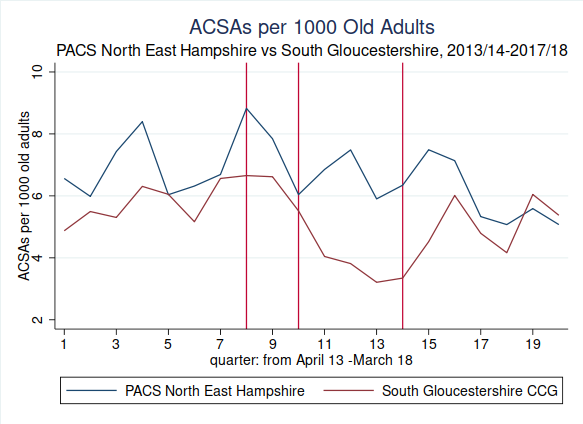
**Figure A2. ACSA rate and HHI trends - matched sample – continued**


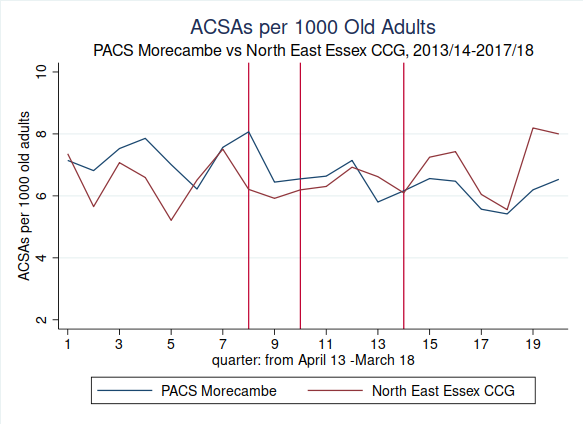


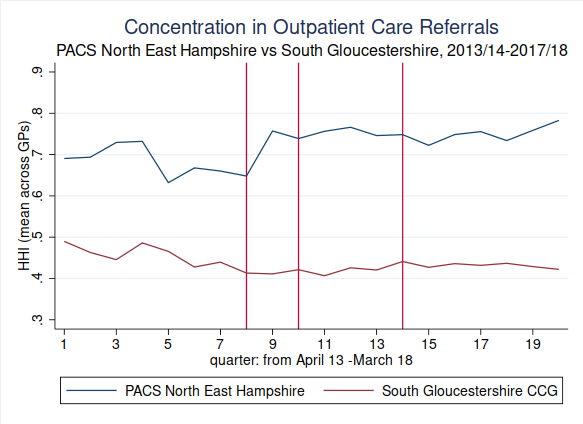

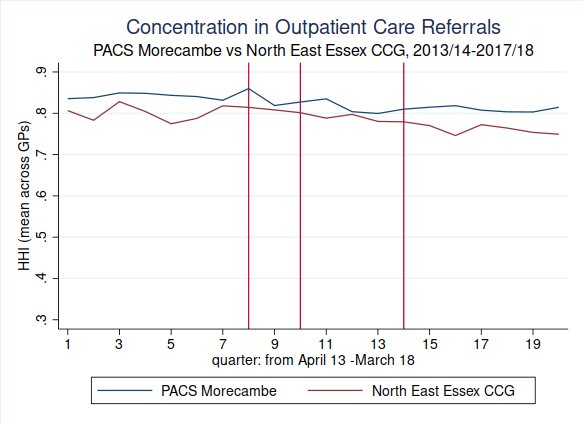


**Figure A2. ACSA rate and HHI trends - matched sample – continued**


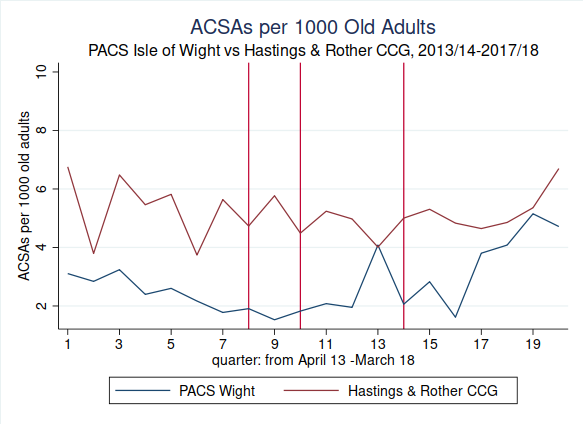


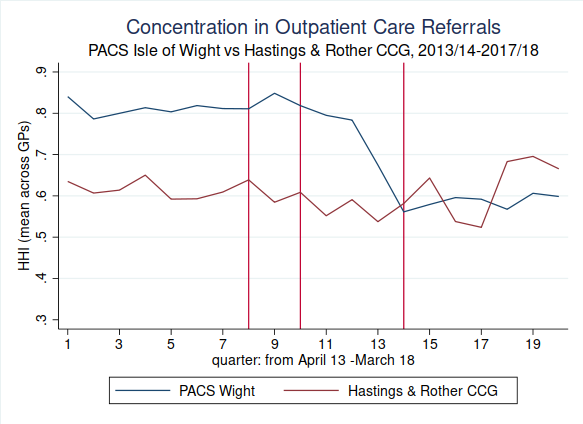

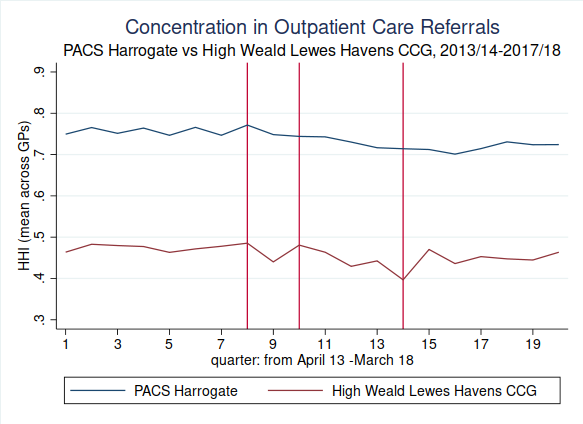

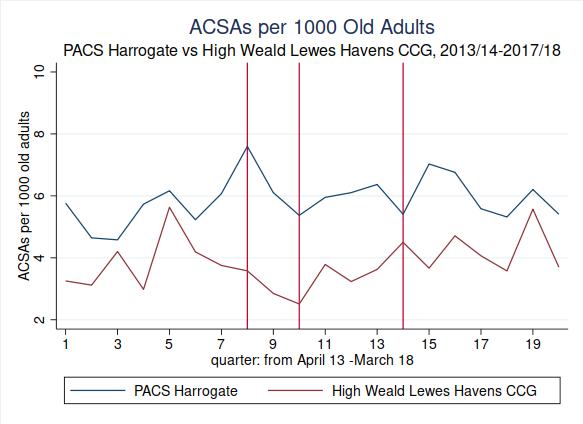

Supplement: Supplementary file 1 — Supplementary Material [file HEC-32-2080-s001.docx]
